# Superior Conjugative Plasmids Delivered by Bacteria to Diverse Fungi

**DOI:** 10.34133/2022/9802168

**Published:** 2022-08-19

**Authors:** Ryan R. Cochrane, Arina Shrestha, Mariana M. Severo de Almeida, Michelle Agyare-Tabbi, Stephanie L. Brumwell, Samir Hamadache, Jordyn S. Meaney, Daniel P. Nucifora, Henry Heng Say, Jehoshua Sharma, Maximillian P. M. Soltysiak, Cheryl Tong, Katherine Van Belois, Emma J. L. Walker, Marc-André Lachance, Gregory B. Gloor, David R. Edgell, Rebecca S. Shapiro, Bogumil J. Karas

**Affiliations:** ^1^Department of Biochemistry, Schulich School of Medicine and Dentistry, The University of Western Ontario, London, ON, Canada, N6A 5C1; ^2^Department of Molecular and Cellular Biology, University of Guelph, Guelph, ON, Canada, N1G 2W1; ^3^Department of Biology, The University of Western Ontario, London, Ontario, Canada, N6A 5B7

## Abstract

Fungi are nature’s recyclers, allowing for ecological nutrient cycling and, in turn, the continuation of life on Earth. Some fungi inhabit the human microbiome where they can provide health benefits, while others are opportunistic pathogens that can cause disease. Yeasts, members of the fungal kingdom, have been domesticated by humans for the production of beer, bread, and, recently, medicine and chemicals. Still, the great untapped potential exists within the diverse fungal kingdom. However, many yeasts are intractable, preventing their use in biotechnology or in the development of novel treatments for pathogenic fungi. Therefore, as a first step for the domestication of new fungi, an efficient DNA delivery method needs to be developed. Here, we report the creation of superior conjugative plasmids and demonstrate their transfer via conjugation from bacteria to 7 diverse yeast species including the emerging pathogen *Candida auris*. To create our superior plasmids, derivatives of the 57 kb conjugative plasmid pTA-Mob 2.0 were built using designed gene deletions and insertions, as well as some unintentional mutations. Specifically, a cluster mutation in the promoter of the conjugative gene *traJ* had the most significant effect on improving conjugation to yeasts. In addition, we created Golden Gate assembly-compatible plasmid derivatives that allow for the generation of custom plasmids to enable the rapid insertion of designer genetic cassettes. Finally, we demonstrated that designer conjugative plasmids harboring engineered restriction endonucleases can be used as a novel antifungal agent, with important applications for the development of next-generation antifungal therapeutics.

## 1. Introduction

The fungal kingdom is exquisitely diverse and home to countless species with profound impacts on ecological nutrient cycling, industrial manufacturing, and health and disease in humans, animals, and plants [1, 2]. Yeast species are amongst the best-studied fungi and include the common yeast *Saccharomyces cerevisiae*, which is a primary fermenter of beer, wine, and bread, and an ubiquitous eukaryotic model system. *S. cerevisiae* is also an important synthetic biology chassis for the production of insulin, vaccine components, and other critical recombinant proteins [3]. The closely related *Saccharomyces boulardii* is a promising probiotic therapeutic, particularly in the context of obesity and type 2 diabetes [4, 5]. Yeasts are also critical components of the human microbiota, including *Candida* species associated with vaginal yeast infections and invasive candidiasis [6], as well as *Malassezia* species with notable associations to Crohn’s disease and pancreatic cancer [7, 8]. The skin-associated yeast *Candida auris* is an emerging fungal pathogen that can cause life-threatening infections and is highly refractory to antifungal drug treatment [9–11]. These diverse and critical roles in health, disease, and industrial manufacturing highlight the importance of studying and manipulating the biology of these key yeast species.

Given the diversity of yeast species and the breadth of niches they inhabit, there is a need to develop improved and innovative methods for DNA transformation in these organisms. Genetic transformation techniques enable the manipulation of genomes of industrially important yeasts, and further promote the ability to target, modify, or damage the genomes of fungal pathogens. Indeed, genetic-editing tools such as CRISPR have a promising role as novel antimicrobial agents due to their ability to specifically target pathogen-associated genes, leading to microbial death, growth inhibition, or targeted deletion of genes involved in antimicrobial resistance or virulence [12–18]. However, laboratory-based transformation protocols typically rely on chemical strategies to promote DNA uptake, which is not broadly applicable for manipulating yeasts in their native environments, such as those inhabiting the microbiome. One innovative strategy to promote the uptake of genetic material in situ is to exploit bacterial conjugation as a viable mechanism to transfer plasmids from bacteria to a recipient microbe via the bacterial type IV secretion system. Previous work has demonstrated the utility of conjugation for transferring plasmids, including those encoding CRISPR-based antimicrobials, between bacterial species, both *in vitro* [19, 20] and *in vivo* in mouse microbiome models [21–23]. While conjugation typically occurs between bacterial species, cross-kingdom conjugation from bacteria to yeast and algae has been demonstrated [24–27]. Despite recently optimized protocols [28, 29], conjugation to yeast still suffers from relatively low conjugation frequency compared to prokaryotic recipients.

Thus, we sought to improve DNA transfer from bacteria to yeast by optimizing the genetic conjugation machinery of the pTA-Mob 2.0 plasmid [28]. This conjugative plasmid is composed of genetic elements required for plasmid maintenance and transfer [30, 31]. Two regions, Tra1 and Tra2, are responsible for the transfer of plasmid DNA. Tra1 harbors the relaxase (*traH*-*J*), primase (*traA*-*G*), and leader (*traK*-*M*) operons, which together coordinate mobilization of the plasmid to the recipient [31]. The relaxase and leader operon encode the relaxosome, a protein complex essential for initial DNA processing during conjugation. Assembly of the protein complex (TraH-J) is initiated by TraJ binding to the 19-bp inverted repeat sequence in the origin of transfer (*oriT*) [32–34]. The interaction of TraI and TraJ, which is stabilized by TraH, then orients the relaxase toward the *nic*-site [34]. After formation of the relaxosome, TraI nicks and covalently binds to the plasmid DNA, ready for transfer to the recipient cell [35, 36]. The efficiency of the nicking reaction is attenuated by the binding of TraK to the *oriT* which orients the plasmid DNA into a more favorable position [33, 37]. TraC1, of the primase operon, is a DNA primase that co-transfers (along with single-stranded binding (SSB) proteins) with the DNA to the recipient cell where it is involved in the restoration of a double-stranded plasmid [38]. The primase operon also includes the TraG protein, which couples DNA processing by the relaxosome to DNA transfer by delivering the protein-DNA complex to the mating pair formation proteins [39, 40]. The Tra2 region contains proteins (TrbB-L and TraF) required for mating pair formation, many of which are associated with the cell membrane. TrbC encodes a peptide responsible for forming the pilus. This peptide undergoes maturation by proteolytic cleavage followed by cyclization by TraF resulting in rigid pili [41, 42]. The pilus allows initial contact between the two cells and enables the transfer of single-stranded plasmid DNA to the recipient cell.

Here, we develop and validate novel plasmids for improved conjugation efficiency between bacteria and diverse yeast species. We demonstrate that a cluster mutation in the relaxase operon, specifically in the *traJ* promoter, significantly improved DNA transfer from bacteria to *S. cerevisiae* and diverse yeasts, including the emerging pathogen *C. auris*. We generate improved, streamlined, and Golden Gate assembly-compatible plasmid derivatives of pTA-Mob 2.0 to enable facile insertion of custom genetic cassettes. Finally, we demonstrate that these designer conjugative plasmids can be used as a novel antifungal reagent, with important applications for the development of next-generation antifungal therapeutics.

## 2. Materials and Methods

### 2.1. Experimental Design

**Figure 1:**
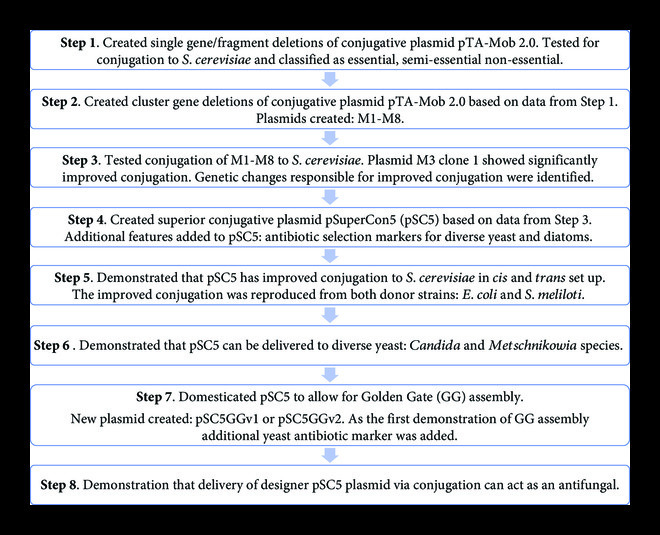
Experiment design. The eight-step flowchart shows experiments and major findings described in this article.

Experimental design is shown in Figure 1.

### 2.2. Microbial Strains and Growth Conditions

*Saccharomyces cerevisiae* VL6−48 (ATCC MYA-3666: *MATα*, *his3-Δ200*, *trp1-Δ1*, *ura3-52*, *lys2*, *ade2-101*, *met14*, *psi + cir^0^*) was grown in yeast media supplemented with ampicillin (100 *μ*g mL^-1^; BioBasic, Cat #: AB0028, Canada) as previously described [43]; or grown with selection on either (1) yeast synthetic complete medium lacking histidine supplemented with adenine hemi-sulfate (Teknova, Inc., Cat #: C7112, USA), (2) yeast synthetic complete medium lacking tryptophan (Teknova, Inc., Cat #: C7131, USA), (3) 2 × YPDA supplemented with nourseothricin (100 *μ*g mL^-1^; Jena BioScience, Cat #: AB-102XL, Germany), or (4) 2 × YPDA supplemented with zeocin (100 *μ*g mL^-1^; Invivogen, Cat #: ant-zn-5p, USA). Solid yeast media contained 2% agar (BioShop Canada Inc., Cat # AGA001.500, Canada). All yeast spheroplast preparation and transformation were performed as previously described [44, 45]. *Escherichia coli* Epi300 (Lucigen Corp., Cat #: LGN-EC300110, USA) was grown as previously described [43] supplemented with appropriate antibiotics (gentamicin (40 *μ*g mL^-1^; BioBasic, Cat #: GB0217, Canada) and chloramphenicol (15 *μ*g mL^-1^; BioBasic, Cat #: CB0118, Canada). Solid media contained 1.5% agar. For transformation of *E. coli*, SOC medium (20 g L^-1^ tryptone, 5 g L^-1^ yeast extract, 0.5 g L^-1^ NaCl, 10 mL 250 mM KCl, 5 mL 2 M MgCl_2_, 20 mL 1 M glucose) was used during the recovery time. Diverse yeasts, *Metschnikowia gruessii* (H53), *Metschnikowia pulcherrima* (CBS 5833), *Metschnikowia lunata* (BS 5946), *Metschnikowia borealis* (SUB 99-207.1), *C. auris*, *Candida tolerans* (UWOPS 98-117.5), *Candida bromeliacearum* (UNESP 00-103), *Candida pseudointermedia* (UWOPS 11-105.1), *Candida ubatubensis* (UNESP 01-247R), and *Candida* aff. *bentonensis* (UWOPS 00-168.1) were grown at 30°C in 2 × YPDA (all diverse yeasts were obtained from Dr. Marc-Andre Lachance collection at Western University except *C. auris* came from https://wwwn.cdc.gov/arisolatebank/ accession number SAMN05379609). *Sinorhizobium meliloti* (Rm4123 R-; obtained from Dr. Finan Lab, McMaster University) was grown at 30°C in LBmc medium (10 g L^-1^ tryptone, 5 g L^-1^ yeast extract, 5 g L^-1^ NaCl, 0.301 g L^-1^ MgSO_4_, and 0.277 g L^-1^ anhydrous CaCl_2_) supplemented with appropriate antibiotics (gentamicin 40 *μ*g mL^-1^ and streptomycin 100 *μ*g mL^-1^; BioBasic, Cat #: SB0494, Canada). Solid media contained 1.5% agar.

### 2.3. Plasmid Construction

#### 2.3.1. PCR Amplification

Plasmid fragments were amplified with GXL polymerase (Takara Bio Inc., Cat #: R050A, Japan) according to the manufacturer’s instructions using annealing temperatures between 50 and 60°C and 25–30 cycles.

#### 2.3.2. Plasmid Assembly in Yeast

Plasmids were assembled in yeast as previously described [28]. Primers for deletion plasmids are listed in Supplemental Table S1 and all primers and templates used to generate other plasmids are listed in Supplemental Table S2: **(1) Single-gene/fragment deletion plasmids**: pTA-Mob 2.0 plasmid was used as a template. Each plasmid was created with nine standard fragments as previously described [28] and two additional fragments designed as shown in Supplemental Figure S1. **(2) Minimized plasmids (M1–8)**: Eight minimized conjugative plasmids (M1–8) were designed based on the results obtained for the pTA-Mob 2.0 deletion plasmids. pTA-Mob 2.0 or M3C1 plasmid was used as a template for PCR fragments listed in Supplemental Table S2. **(3) M3C1_F1 - F5 hybrid plasmids**: These plasmids were assembled by swapping the fragments between M3C1 and M3C2. **(4) pTA-Mob 2.0 Tp and pTA-Mob 2.0 To**: Primer-mediated mutagenesis was performed to introduce each mutation (Tp- in *traJ* promoter and To- in *traJ* ORF) into pTA-Mob 2.0. **(5) Superior conjugative plasmid (pSC5)**: The pSC5 plasmid was derived from M3C1 plasmid by the addition of two versions of Nourseothricin N-acetyl transferase (*NAT*) genes which provide resistance for nourseothricin antibiotic. The first version was amplified from a plasmid pTA-Mob-NAT (unpublished, Karas lab) allowing selection in diatoms and referred to as *dNAT*; and the version was amplified from pGMO1 (unpublished, Karas lab), which contained an alternative genetic code for selection in diverse yeasts as previously described [46] and referred to as *yNAT*. The remaining fragments of the pSC5 plasmid were amplified from M3C1 as previously described [28]. **(6) pTA-Mob 2.1/pSC5.1**: These two plasmids were created lacking the second copy of *traJ* located in the vector backbone. **(7) Domesticated pSC5 (pSC5GGv1/v2**): Two BsaI cut sites located within the *fcpD* promoter and *traC1* ORF were removed from pSC5 using primer-mediated mutagenesis. An RFP landing pad, consisting of a monomeric Red Fluorescent Protein (mRFP) gene driven by an arabinose-inducible pBAD promoter and a terminator, was amplified from pAGE2.0-i (unpublished, Karas lab) using primers designed with new BsaI cut sites and homology either to directly downstream the native I-SceI restriction site (pSC5GGv1) or within the *HIS3*/*CEN6*/*ARS4* element of the vector backbone (pSC5GGv2).

#### 2.3.3. Golden Gate Assembly

(1) Golden Gate (GG) assembly. For GG assembly, 20 fmol of plasmid and insert were mixed in a 15 *μ*L reaction with 1.0 *μ*L T4 DNA ligase (New England BioLabs, Inc., Cat #: M0202L, USA) and 0.5 *μ*L BsaI-HF V2 (New England BioLabs, Inc., Cat #: R3733S, USA), using the following conditions: 10 cycles of 37°C for 5 minutes and 16°C for 10 minutes followed by incubation at 37°C for 5 minutes, 80°C for 10 minutes, and infinite hold at 12°C. Primers are listed in Supplemental Table S2. (2) pSC5GGv1_ShBle. The zeocin (ShBle) resistance marker cassette was amplified with flanking BsaI cut sites from pRS32 (unpublished, Shapiro Lab) and GG assembly was performed with pSC5GGv1. The primers used are listed in Supplemental Table S2. (3) pSC5-toxic1, pSC5-toxic2, and pSC5-toxic3. Three versions of toxic plasmids to kill yeast cells were created, one with an *Acholeplasma laidlawii* toxic gene (ACL0117) [47] and two versions with the restriction enzyme HindII. The *A. laidlawii* and HindII cassettes both contained an *ACT1* yeast intron, flanking BsaI cut sites, and either an *A. laidlawii* or HindII toxic gene. The *A. laidlawii* toxic gene cassette was amplified in three fragments: the *ACT1* yeast intron from *S*. *cerevisiae*VL6-48 gDNA and the toxic gene in two halves from *A. laidlawii* PG-8A gDNA with primers listed in Supplemental Table S2. The *A. laidlawii* toxic gene cassette was then constructed through a hierarchical GG assembly. First, the *ACT1* yeast intron and the second half of the *A. laidlawii* toxic gene were assembled by GG assembly, and then 1 *μ*L of the product was used as a template for PCR amplification of the joined fragments. Next, GG assembly was performed with 20 fmol of the PCR product with the first half of the toxic gene, and the complete toxic gene cassette was PCR amplified. The fully constructed cassette was then mixed with pSC5GGv1, and GG assembly was performed. The HindII cassette split by the *ACT1* intron was flanked by the *URA3* promoter/terminator and was synthesized (BioMatik, Canada), then PCR amplified and used in GG assembly with pSC5GGv1 or pSC5GGv2

### 2.4. Plasmid Analysis

**Screening in yeast**. Following yeast assembly of the pTA-Mob 2.0 deletion plasmids, 20 individual yeast colonies were passed twice on solid media lacking histidine, and DNA was isolated and screened by multiplex PCR using the Qiagen Multiplex Kit (Qiagen, Inc., Cat #: 206143, Germany) according to the Qiagen Multiplex PCR Handbook. For all other plasmids following yeast assembly, colonies were pooled rather than individually screened. **Transformation to *E. coli***. Total DNA was isolated as previously described [24]. Isolated DNA (0.5**–**2 *μ*L) was added to *E. coli* Epi300 electro-competent cells (40 *μ*L) and electroporated using the Gene Pulser Xcell Electroporation System (2.5 kV voltage, 25 *μ*F capacitance, and 200 *Ω* resistance). Following a recovery in 1 mL of SOC medium for 1 hour at 37°C (225 RPM), a 100**–**250 *μ*L aliquot of the transformants was plated on LB medium supplemented with gentamicin (40 *μ*g mL^-1^). The cells transformed with Golden Gate-compatible plasmids (pSC5GGv1/v2, pSC5-toxic1, pSC5-toxic2, and pSC5-toxic3) were instead plated on LB plates containing gentamicin (40 *μ*g mL^-1^) and arabinose (100 *μ*g mL^-1^). **Screening in *E. coli***. For Golden Gate-compatible plasmids, white colonies were then screened by multiplex PCR for insertion of the cassette of interest. For all other assembled plasmids, the transformed *E. coli* was pooled and conjugated to *S. cerevisiae*, and DNA was re-isolated and transformed back to *E. coli* to focus screening on functional conjugative plasmids. Once in *E. coli*, all plasmids were genotypically screened using multiplex PCR and restriction enzyme digest analysis. **Sequencing**. The plasmids, pTA-Mob 2.0 Tp/To, underwent Sanger DNA sequencing (London Regional Genomics Centre at Robarts Research Institute) to ensure the introduction of the correct mutations using the primers listed in Supplemental Table S2. Selected plasmids were sequenced at CCIB DNA Core at Massachusetts General Hospital or at the Western University sequencing facility.

### 2.5. Conjugation

Both donor (*E. coli*) and recipient (*S. cerevisiae*) strains were prepared and frozen prior to conjugation experiments. For *E. coli* strains, saturated overnight cultures inoculated with a single colony were diluted to OD_600_ of 0.1 in 50 mL of LB medium supplemented with appropriate antibiotics (Table 1) and grown until an OD_600_ of 1.0 was reached. The cells were pelleted (3,000 × RCF, 15 minutes) in a 50 mL Falcon tube and resuspended in 500 *μ*L ice-cold 10% glycerol. Then, 100 *μ*L aliquots in Eppendorf tubes were frozen in a -80°C ethanol bath and stored at -80°C. For *S. cerevisiae* recipient strain preparation, a culture was started from a single colony and grown in 5 mL of 2 × YPDA medium supplemented with ampicillin (100 *μ*g mL^-1^) for 7 hours. After, this culture was diluted in 50 mL of 2 x YPDA medium supplemented with ampicillin (100 *μ*g mL^-1^) and grown until an OD_600_ of 3.0 was reached (~17 hours). The cells were pelleted (3,000 × RCF, 5 minutes) in a 50 mL Falcon tube and resuspended in 1 mL of ice-cold 10% glycerol. Then, 250 *μ*L aliquots in Eppendorf tubes were frozen in a -80°C ethanol bath and stored at -80°C.

**Table 1:** Plasmids used in this study. *aacC1* provides resistance to gentamicin; *cat* to chloramphenicol; *bla* to ampicillin; *yNAT* to nourseothricin in diverse yeast; *ShBle* to zeocin in diverse yeasts. *HIS3* is required for the histidine biosynthesis; *URA3* for uracil biosynthesis; *TRP1* for tryptophan biosynthesis in *S. cerevisiae*. ^†^ - duplicated copy that is present in the plasmid backbone.

Plasmid	Plasmid size (kb)	*E. coli* marker	Yeast marker	Citation
**pTA-Mob 2.0**	57	*aacC1*	*HIS3*/*URA3*	Soltysiak et al. (2019)
**pAGE1.0**	18	*cat*	*HIS3*	Brumwell et al. (2019)
**pAGE2.0.T**	19	*cat*	*TRP1*	This study
**pAGE2.0-i**	20	*cat*	*HIS3*	This study
**pAGE2.0-iTraJ**	20	*cat*	*HIS3*	This study
**pRS32**	11	*bla*	*ShBle*	Shapiro *et al.* (unpublished)
**M1C1:** pTA-Mob 2.0 *Δ*TrbO-FiwA	54	*aacC1*	*HIS3*/*URA3*	This study
**M1C2:** pTA-Mob 2.0 *Δ*TrbO-FiwA	54	*aacC1*	*HIS3*/*URA3*	This study
**M2C5:** pTA-Mob 2.0 *Δ*KlaC-KleA	53	*aacC1*	*HIS3*/*URA3*	This study
**M3C1:** pTA-Mob 2.0 *Δ*IstA-TraB	52	*aacC1*	*HIS3*	This study
**M3C2:** pTA-Mob 2.0 *Δ*IstA-TraB	52	*aacC1*	*HIS3*	This study
**M4C16:** pTA-Mob 2.0 *Δ*IstA-TraE	46	*aacC1*	*HIS3*	This study
**M4C19:** pTA-Mob 2.0 *Δ*IstA-TraE	46	*aacC1*	*HIS3*	This study
**M5C1:** pTA-Mob 2.0 *Δ*TrbN-TraE	40	*aacC1*	*HIS3*	This study
**M5C3:** pTA-Mob 2.0 *Δ*TrbN-TraE	40	*aacC1*	*HIS3*	This study
**M6C2:** pTA-Mob 2.0 *Δ*TrbN-TraE, *Δ*KlaC-KleA	35	*aacC1*	*HIS3*	This study
**M6C4:** pTA-Mob 2.0 *Δ*TrbN-TraE, *Δ*KlaC-KleA	35	*aacC1*	*HIS3*	This study
**M7C2:** pTA-Mob 2.0 *Δ*TrbN-TraE, *Δ*TrfA-TraJ^†^	37	*aacC1*	*HIS3*	This study
**M8C8:** pTA-Mob 2.0 *Δ*TrbM-TraE, *Δ*KlaC-KleA, *Δ*TrfA-TraJ^†^	31	*aacC1*	*HIS3*	This study
**M8C10:** pTA-Mob 2.0 *Δ*TrbM-TraE, *Δ*KlaC-KleA, *Δ*TrfA-TraJ^†^	31	*aacC1*	*HIS3*	This study
**M3C1_F1:** pTA-Mob 2.0 *Δ*IstA-TraB	52	*aacC1*	*HIS3*	This study
**M3C1_F2:** pTA-Mob 2.0 *Δ*IstA-TraB	52	*aacC1*	*HIS3*	This study
**M3C1_F3:** pTA-Mob 2.0 *Δ*IstA-TraB	52	*aacC1*	*HIS3*	This study
**M3C1_F4:** pTA-Mob 2.0 *Δ*IstA-TraB	52	*aacC1*	*HIS3*	This study
**M3C1_F5:** pTA-Mob 2.0 *Δ*IstA-TraB	52	*aacC1*	*HIS3*	This study
**pTA-Mob 2.0 Tp**	56	*aacC1*	*HIS3*/*URA3*	This study
**pTA-Mob 2.0 To**	56	*aacC1*	*HIS3*/*URA3*	This study
**pSC5:** pTA-Mob 2.0 *Δ*IstA-TraB	56	*aacC1*	*HIS3*/y*NAT*	This study
**pTA-Mob 2.1:** pTA-Mob 2.0 *Δ*TraJ^†^	56	*aacC1*	*HIS3*/*URA3*	This study
**pSC5.1:** pTA-Mob 2.0 *Δ*IstA-TraB, *Δ*TraJ^†^	55	*aacC1*	*HIS3*/y*NAT*	This study
**pSC5GGv1:** pTA-Mob 2.0 *Δ*IstA-TraB, +mRFP	57	*aacC1*	*HIS3*/y*NAT*	This study
**pSC5GGv2:** pTA-Mob 2.0 *Δ*IstA-TraB, +mRFP	57	*aacC1*	*HIS3*/y*NAT*	This study
**pSC5GGv1_ShBle:** pTA-Mob 2.0 *Δ*IstA-TraB	57	*aacC1*	*HIS3*/yNAT/*ShBle*	This study
**pSC5-toxic1:** pTA-Mob 2.0 *Δ*IstA-TraB, +ACL0117	59	*aacC1*	*HIS3*/y*NAT*	This study
**pSC5-toxic2:** pTA-Mob 2.0 *Δ*IstA-TraB, +HindII	57	*aacC1*	*HIS3*/y*NAT*	This study
**pSC5-toxic3:** pTA-Mob 2.0 *Δ*IstA-TraB, +HindII	57	*aacC1*	*HIS3*/y*NAT*	This study

On the day of conjugation, conjugation plates (20 mL, 1.8% agar, 10% LB medium, complete minimal glucose broth lacking histidine) were dried for 30 minutes. Aliquots of the donor (*E. coli*) and recipient (*S. cerevisiae*) strains were removed from the freezer and thawed on ice for approximately 20 minutes. Next, 50 *μ*L of *S. cerevisiae* was added to the 100 *μ*L of *E. coli* and mixed by gentle pipetting before being transferred to the plate and spread evenly. Alternatively, when the yeast toxic plasmids were being tested, 10 *μ*L of the recipient *S. cerevisiae* strain was used. Once dried, the plates were incubated at 30°C for 3 hours, or 12 hours when wild yeast strains were used as the recipient. The plates were scraped with 2 mL of sterile double-distilled water (sddH_2_O), mixed by vortexing for 5 seconds, and 100 *μ*L plated on respective selection media (25 mL, 2% agar supplemented with ampicillin 100 *μ*g mL^-1^) listed in Table 1. In the case of wild yeast strains, they were plated on 1×YPDA medium supplemented with nourseothricin (100 *μ*g mL^-1^) and two technical replicates of each dilution (10^0^–10^-1^) were plated on selective plates. For experiments evaluating conjugation of pSC5 in *cis* and *trans*, dilution series of 10^0^–10^-2^ were generated and plated on selective media while dilution series of 10^-4^–10^-7^ were generated and plated on non-selective medium (1 × YPDA supplemented with ampicillin 100 *μ*g mL^-1^); and two technical replicates were plated for each dilution.

### 2.6. RNA Isolation and Quantitative Reverse Transcriptase-Polymerase Chain Reaction

For RNA isolation, the *E. coli* strains carrying conjugative plasmids pTA-Mob 2.1 and pSC5.1 were grown in LB medium supplemented with gentamicin (40 *μ*g mL^-1^) overnight at 37°C shaking at 225 RPM. In the morning, RNA was isolated as previously described [43]. Following DNase treatment with TURBO DNA-*free*™ Kit (Invitrogen Cat #: AM1907, USA), the RNA concentration and the integrity was verified [43].

cDNA was prepared from 500 ng of RNA using the High-Capacity cDNA Reverse Transcription Kit (Applied Biosystems, Cat #: 4368814, USA). qRT-PCR was performed using six biological and three technical replicates, on a ViiA7 system of QuantStudio Real-Time PCR System (Applied Biosystems, USA) using the SYBR™ Select Master Mix (Applied Biosystems, Cat #: 472908, USA) under the following conditions: 50°C for 2 minutes, 95°C for 2 minutes followed by 40 cycles of: 95°C for 1 second, 60°C for 30 seconds. Expression levels were normalized against two reference genes (*rrsA* and *cysG*) as previously described [29]. Primer sequences used for the qRT-PCR expression analyses are listed in Supplemental Table S2.

### 2.7. Statistical Analysis

The pairwise comparisons between groups were made using Student’s t-test with either equal or unequal variance based on the result of an F-test. Data were expressed as either ±95% confidence interval (CI) or as mean ± standard error of the mean (SEM) or as mean ± standard deviation of at least three biological replicates. The tests were considered statistically significant when P<0.05 (${}^{*}$), P<0.01 (${}^{**}$), or P<0.001 (${}^{***}$).

## 3. Results

### 3.1. Development of Streamlined Conjugation Plasmids

**Figure 2:**
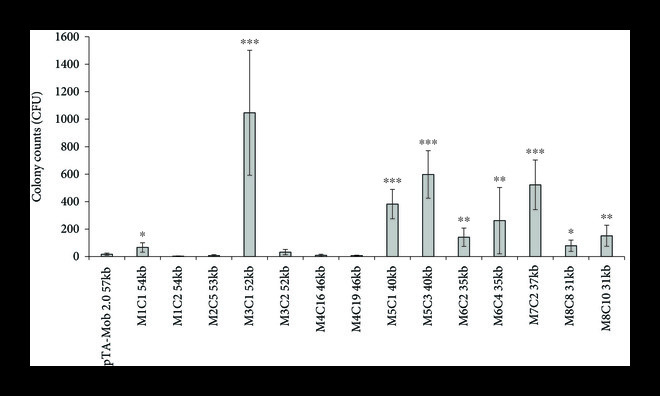
Transfer of minimized plasmids from *E. coli* to *S. cerevisiae* via conjugation. The pTA-Mob 2.0 plasmid was used as a template to create versions M1–M4. M3C1 was used as a template to create M5–M8. When available, two clones of the same plasmid were tested (e.g., M1 C1 and C2). Error bars represent ±95% confidence interval (Student’s t-test was used to carry out pairwise comparisons between pTA-Mob 2.0 (control) and minimized versions of pTA-Mob 2.0: ${}^{*}P<0.05$, ${}^{**}P<0.01$; ${}^{***}P<0.001$). N = 9 for all strains except N = 27 for pTA-Mob 2.0.

As a first step (Figure 1) toward creating an optimized and minimized conjugative plasmid for yeast, 55 single genes or small genetic regions were individually deleted from our previously established trans-kingdom conjugation plasmid, pTA-Mob 2.0 [28] (Supplemental Figure S1, Supplemental Table S1). To validate these plasmid variants, up to two clones of each were tested for conjugation from *E. coli* to *S. cerevisiae*, and the genes/regions deleted were classified as essential (no conjugation), semi-essential (decreased conjugation), or non-essential (near wild type conjugation) for bacteria-to-yeast conjugation (Supplemental Table S3). Based on this data, four streamlined plasmids were created where clusters of non-essential genes were simultaneously removed (plasmids M1–M4, Table 1, Supplemental Table S2). Plasmids M1–M4 were then conjugated from *E. coli* to yeast, and we observed a significant increase in successful conjugation efficiency for plasmid M3 clone 1 (M3C1), monitored by yeast colony formation on selective media (Figure 2). Sequencing both M3 clones, M3C1 and M3C2, revealed multiple mutations in each clone, which are likely responsible for the increase in conjugation efficiency for M3C1 (Supplemental Table S4).

**Figure 3:**
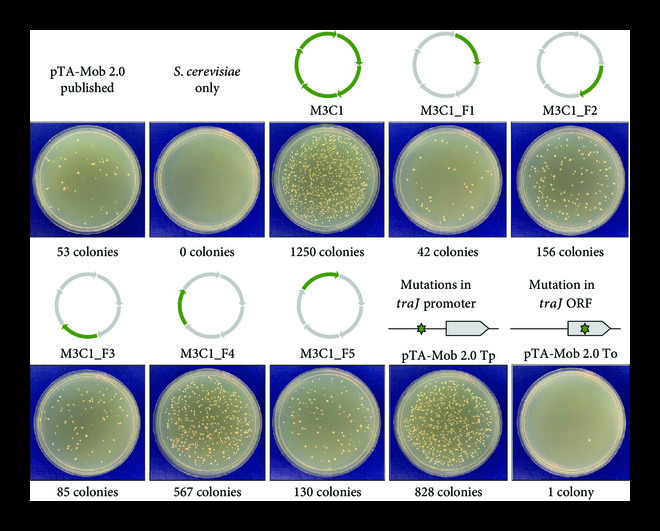
Identification of mutations in M3C1 responsible for improved conjugation to *S. cerevisiae*. *S. cerevisiae* transconjugant colony formation on minimal plates lacking histidine following conjugation of control: pTA-Mob 2.0, M3C1; hybrid: M3C1/M3C2; or mutated pTA-Mob 2.0 Tp/To plasmids from *E. coli*. Plasmid schematics for M3C1_F1–F5 generated by swapping PCR-amplified fragments from M3C1 into M3C2 are shown. Green arrows represent fragments originating from M3C1 and gray arrows represent fragments originating from M3C2. Mutations in pTA-Mob 2.0 Tp/To plasmids are indicated by green stars in the schematic of *traJ* (Tp, a cluster of mutations; and To, a single mutation). Transconjugant colony counts are reported below the plate images.

To identify which mutations in M3C1 were responsible for the increased conjugation efficiency, we performed a fragment swapping experiment between M3C1 and M3C2 to produce five hybrid plasmids M3C1_F1–F5 (Figure 3). Each hybrid plasmid was created from four fragments that were amplified from M3C2 and one fragment from M3C1. Hybrid plasmid M3C1_F4 (fragment 4 originated from M3C1) had the closest conjugation efficiency compared to M3C1 (Figure 3). There were two mutated regions in fragment 4 of M3C1: a cluster of mutations in the promoter of *traJ*, and a single mutation in the open reading frame (ORF) of *traJ* (Supplemental Table S4). To validate which mutation(s) contributed to the increased conjugative phenotype, the promoter or ORF *traJ* mutations were introduced into pTA-Mob 2.0 and tested for conjugation efficiency. Only the mutations in the promoter region of *traJ* improved conjugation efficiency (Figure 3). Additionally, we continued to minimize M3C1 by creating new plasmids with additional non-essential genes removed to obtain M5–M8 plasmids (Figure 2). All M5–M8 minimized plasmids still produced more colonies when conjugated to yeast as compared to the original pTA-Mob 2.0 (Figure 2).

### 3.2. Creation of Superior Conjugative Plasmid pSuperCon5

**Figure 4:**
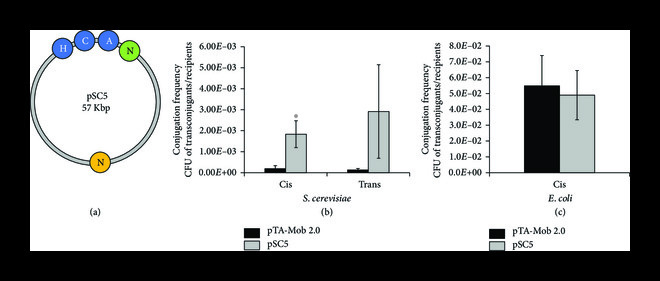
Creation and analysis of the pSC5 conjugative plasmid. (a) Schematic of pSC5 plasmid map. N: nourseothricin resistance gene encoded with the standard code for diatom (green) or the alternative yeast nuclear code for *Candida*/*Metschnikowia* (orange; note: also correctly translated in *S. cerevisiae*), HCA—*HIS3*, *CEN6*, and *ARS4* for selection, replication, and maintenance in *S. cerevisiae*. b) Conjugation frequency of pSC5 (gray) compared to pTA-Mob 2.0 (black) in either a *cis* or *trans* setup from *E. coli* to *S. cerevisiae*. (c) Bacterial conjugation frequency of pSC5 from *E. coli* to *E. coli* in a *cis-*configuration. Error bars represent ±95% confidence interval (Student’s t-test: ${}^{*}P<0.05$, ${}^{**}P<0.01$, ${}^{***}P<0.001$). N = 3 for *cis*- and N = 4 for *trans*-experiment in *S*. *cerevisiae* and N = 3 for *cis*-experiment in *E*. *coli.*

Based on the identified *traJ* promoter mutations, we created the pSuperCon5 (pSC5) plasmid with additional elements to enable the delivery of our improved conjugative plasmids to diverse yeast and diatoms. The pSC5 plasmid was built based on M3C1 and contains two copies of the nourseothricin resistance gene (y*NAT* and *dNAT*): one optimized for selection in diverse yeast and one for diatoms (Figure 4(a)). Conjugation frequency for pSC5 from *E. coli* to *S. cerevisiae* was increased approximately 10- or 23-fold compared to pTA-Mob 2.0 when tested in *cis* (mobilizing itself) or *trans* (mobilizing another plasmid), respectively (Figure 4(b), Supplemental Table S5 & S6). No significant difference in conjugation frequency was observed when plasmids were transferred between *E. coli* strains (Figure 4(c), Supplemental Table S5 and S6).

In order to more precisely evaluate the frequency of bacteria-to-yeast conjugation, we performed additional experiments to monitor the effect of conjugation plasmid-containing *E. coli* on yeast viability. Cells from *E. coli*-to-yeast conjugation experiments were plated on non-selective yeast medium supplemented with ampicillin to inhibit *E. coli* growth. More yeast colonies grew when pSC5 was used versus pTA-Mob 2.0 (Supplemental Table S7), indicating that *E. coli* carrying pSC5 has fewer adverse effects on yeast when they are co-cultured. To determine if the same effect could be observed when different donor cells were used, we performed conjugation with *Sinorhizobium meliloti* as a donor, as it was previously shown to conjugate to yeast [25]. Similarly, a higher number of yeast colonies grew on non-selective plates when *S. meliloti* harboring pSC5 was used when compared to pTA-Mob 2.0 (Supplemental Table S8). In addition, a higher number of colonies on selective plates were observed following conjugation of pSC5 from *S. meliloti* to *S. cerevisiae* compared to pTA-Mob 2.0 (Supplemental Figure S2, Supplemental Table S9).

Additional experiments will need to be performed to determine if there is a link between the increased number of yeast colonies on non-selective/selective plates and the lower expression of *traJ* (Supplemental Figure S3) in plasmids carrying the promoter mutation.

### 3.3. Conjugation to Diverse Yeast Species

**Figure 5:**
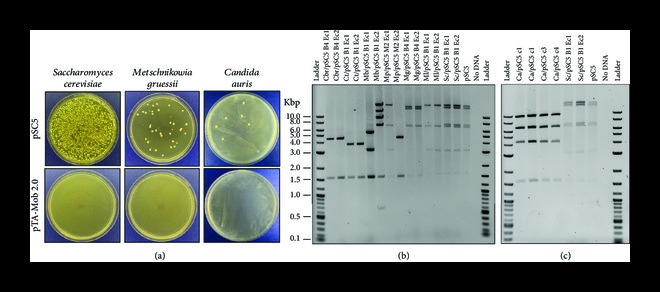
Conjugation of pSC5 to wild yeast strains. (a) pSC5 conjugated to *S. cerevisiae* and wild yeasts *M. gruessi* and *C. auris* were plated on YPD plates supplemented with NTC (100 *μ*g mL^-1^). (b, c) Diagnostic double restriction enzyme digestion (EcoRI-HF and AgeI-HF) of pSC5 plasmid rescued from yeast strains. The expected band sizes for pSC5 are 20,808, 15,855, 7,314, 6,848, 3,189, 1,610, and 6 bp. Cbr: *C. bromeliacearum*; Ct: *C. tolerans*; Mb: *M. borealis*; Mp: *M. pulcherrima*; Mg: *M. gruessi*; Ml: *M. lunata*; *Ca*: *C. auris*; and Sc: *S. cerevisiae.* Ladder: NEB 2-log ladder.

The significantly improved frequency of conjugation with the pSC5 plasmid suggests it may be effective for conjugation beyond a standard laboratory strain of *S. cerevisiae* and may have utility in transferring DNA from bacteria to diverse yeast species. To test the ability of pSC5 to transfer DNA to diverse yeast strains, we selected four *Metschnikowia* and six *Candida* species as conjugative recipients. Previously, we have demonstrated that small DNA fragments (y*NAT* selection marker) can be delivered to most of these species by electroporation [46]. Conjugation to these diverse yeasts was performed with the same protocol used for *S. cerevisiae*, modified to allow for selection on complete yeast media containing antibiotics. Transconjugant colonies were obtained for all species (Figure 5(a), Supplemental Figure S4, S5), and 1–8 colonies for each species were genotyped by PCR for the presence of the y*NAT* marker. Of the 10 species tested, seven tested positive by PCR for the presence of the y*NAT* marker, suggesting successful conjugation had occurred (Supplemental Figure S6). A plasmid rescue experiment, where total yeast genomic DNA is electroporated into *E. coli*, was performed for selected colonies for each of the seven species, as well as *S. cerevisiae.* pSC5 plasmids from the seven yeast species were successfully recovered in *E. coli*; however, all except those recovered from *S. cerevisiae* showed rearrangements when diagnostic restriction enzyme digestion was performed (Figures 5(b) and 5(c)). Furthermore, only the plasmids recovered from *S. cerevisiae* were still able to conjugate (Supplemental Figure S7).

### 3.4. Domestication of pSC5 for Golden Gate Assembly

**Figure 6:**
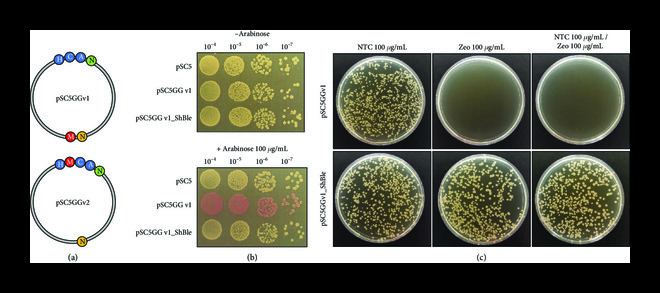
Development of pSC5 plasmid compatible with GG assembly. (a) Two versions of the pSC5 plasmid were created. Version 1 (pSC5GGv1) has the mRFP landing pad located in the middle of the plasmid, and version 2 (pSC5GGv2) has the mRFP located between yeast elements *HIS3* and *CEN6*-*ARS4*. (b) Example of GG assembly to insert a gene of interest (*ShBle*) into pSC5GGv1. pSC5: original plasmid; pSCGGv1: domesticated plasmid; pSC5GGv1_ShBle: a selected *E. coli* colony with *ShBle* inserted grown on LB plates supplemented with gentamicin (40 *μ*g mL^-1^). (c) Conjugation of pSC5GGv1 and pSC5GGv1_ShBle to *S. cerevisiae*. NTC: nourseothricin; Zeo: zeocin.

Next, we sought to modify the pSC5 plasmid to make it readily amenable to cloning for easy incorporation of any desired DNA fragment, to facilitate downstream applications of bacterial-to-yeast conjugation. To this end, we eliminated existing BsaI restriction sites from pSC5 and created a single BsaI-based Golden Gate cloning-compatible site to enable efficient plasmid manipulation [48, 49]. In addition, we incorporated a landing pad with mRFP driven by an arabinose-inducible promoter (Figure 6(a)). In this modified plasmid, Golden Gate assembly can readily be used to replace the mRFP gene with any gene of interest, allowing for an easy visual screen for correct gene insertion events (white versus red bacterial colonies).

To validate this system, we inserted a second antibiotic marker (*ShBle*) for yeast into pSC5GGv1 (Figure 6(a)) to create pSC5GGv1_ShBle, which provides resistance to zeocin. White bacterial colonies were selected (Figure 6(b)) and genotyped with diagnostic multiplex PCR and restriction digest (data not shown). These validated colonies were conjugated to *S. cerevisiae* and tested for survival on single or double antibiotic selection (Figure 6(c)). Successful exconjugants which received pSC5GGv1_ShBle were able to grow on media supplemented with zeocin, nourseothricin, or both (Figure 6(c)).

### 3.5. Proof of Concept for Conjugation-Mediated Delivery as an Antifungal

**Figure 7:**
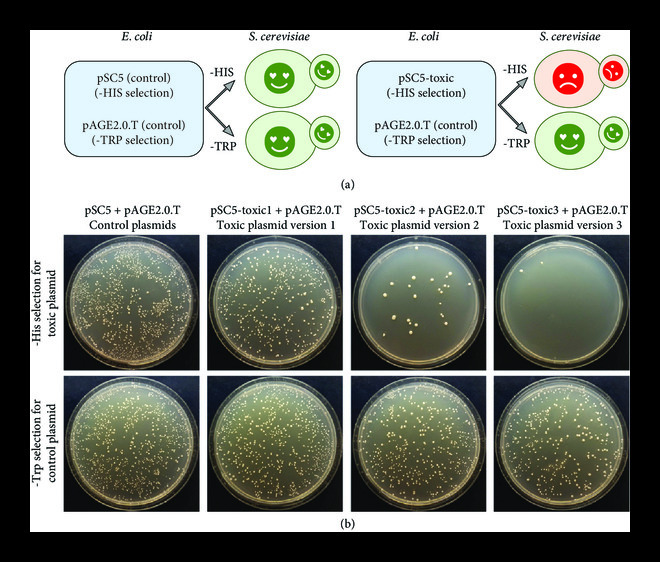
Conjugation-based antifungal. (a) Diagram illustrating the predicted outcomes on *S. cerevisiae* colony formation following delivery of various plasmids via bacterial conjugation. The control strain (left) harboring pSC5 and pAGE2.0.T selected on synthetic yeast media lacking either histidine or tryptophan will survive. Conversely, the experimental strain (right) harboring pSC5-toxic gene plasmid and pAGE2.0.T when selected on synthetic yeast medium lacking histidine will die. (b) Assay for *S. cerevisiae* colony formation on -HIS or -TRP media following bacterial conjugation with three toxic plasmids. pSC5-toxic1: partially toxic gene identified in *A. laidlawii* inserted in pSC5GGv1; pSC5-toxic2: *H. influenzae* HindII restriction gene inserted in pSC5GGv1; pSC5-toxic3: *H. influenzae* HindII restriction gene inserted in pSC5GGv2.

To demonstrate that pSC5-based conjugating plasmids could be used as an antifungal, we developed a system where each donor *E. coli* strain carried two plasmids: a control plasmid (pAGE2.0.T) that can be selected on media lacking tryptophan and either pSC5 or pSC5-toxic gene plasmid that can be selected on media lacking histidine (Figure 7(a)). We used Golden Gate assembly to create three pSC5-toxic plasmids, each carrying a gene that should be partially or fully toxic to yeast. To prevent toxicity in *E. coli*, we inserted a yeast *ACT1* intron [50] into each toxic gene. Next, we cloned the *A. laidlawii* toxic gene [47] into pSC5GGv1 to generate pSC5-toxic1, or an *Haemophilus influenzae* HindII restriction gene into pSC5GGv1 and pSC5GGv2 to generate pSC5-toxic2 and pSC5-toxic3, respectively. The pSC5 and pSC5-toxic gene plasmids can act in *cis*, mobilizing themselves, as well as in *trans*, mobilizing the control plasmid pAGE2.0.T. Using a control *E. coli* strain carrying plasmids pSC5 and pAGE2.0.T, we observed a similar colony number on both selection plates. For conjugation with pSC5-toxic gene plasmids, substantially fewer colonies grew on minimal media lacking histidine. The most substantial difference in yeast colony formation was with donor *E. coli* carrying pSC5-toxic3 (Figure 7(b), Supplemental Table S10). This provides a proof of concept that bacteria-to-yeast conjugation can be used to effectively deliver plasmid-based antifungals.

## 4. Discussion

Conjugation-based techniques, such as the one described here, provide a unique and functional method to deliver plasmids between microbial species *in vitro* and *in vivo*. While there are innumerable possible applications for these systems, many have focused on the use of plasmid-encoded CRISPR-based genetic manipulation systems to modify the genomes of the recipient microbes [19, 23, 51]. Indeed, CRISPR-based gene targeting and manipulation systems offer a breadth of applications that can be paired with conjugation (or other methods of DNA delivery, such as phage transduction) to achieve desired manipulation of a target microbial population. The majority of this work to date has focused on bacterial species. For instance, CRISPR-based systems have been used to induce lethal DNA damage in key bacterial pathogens, including *E. coli*, *Staphylococcus aureus*, and *Clostridium difficile*, to effectively eradicate unwanted bacterial populations, including drug-resistant bacteria [13, 14, 52], and specific pathogenic species or sub-populations [13, 16–19, 53, 54]. In addition to directly killing bacterial populations, CRISPR systems can also be applied to modify virulence determinants to erode microbial pathogenicity [53, 55], or alter drug-resistance genes to restore antimicrobial susceptibilities [15, 23, 56–60]. To enable the application of these CRISPR systems *in vivo*, many have relied on the use of bacterial conjugation or phage transduction as methods to deliver the relevant CRISPR components [13, 17, 21–23, 61]. While this has been effective for delivery to bacterial strains, it has limited the applications in fungi, which lack well-established tools for conjugation or virus-based gene delivery [62, 63].

To address the bottleneck of improving DNA delivery to yeast, we performed experiments to optimize the conjugative plasmid pTA-Mob 2.0 [28]. We first evaluated whether plasmid derivatives with targeted deletions of the conjugative plasmid could improve DNA transfer to *S. cerevisiae*. After testing 57 single-gene and four cluster-gene deletion plasmids, one with superior conjugative properties (M3C1) was identified. Sequence analysis of M3C1 revealed that in addition to the designed deletions, M3C1 had unintended mutations that were likely introduced during PCR amplification or plasmid assembly. The mutations responsible for improved conjugation to *S. cerevisiae* were narrowed down to the promoter region of *traJ* (Tp) using a fragment swapping experiment. Following this discovery, five derivative plasmids of M3C1 were built containing the Tp mutation: four minimized versions (M5–M8) and pSuperCon5 (pSC5, containing selectable markers for diverse yeast species [46] and diatoms [24]). Each derivative plasmid of M3C1, including the smallest 31 kb plasmid M8, outperformed the original 57 kb pTA-Mob 2.0 plasmid when tested for DNA transfer to *S. cerevisiae*. Notably, using pSC5 compared to pTA-Mob 2.0, we observed an increase in conjugation to *S. cerevisiae* 10- or 23-fold in either a *cis* or *trans* setup, respectively. Yet, no increase in plasmid transfer was observed when pSC5 was conjugated between *E. coli* strains. This improved conjugation to *S. cerevisiae* could be partially explained by the increased *S. cerevisiae* viability during the co-culture conjugation step when plasmids harboring the Tp mutation are used. The same effect was also observed when *S. meliloti* was used as a conjugative donor, suggesting the mechanism may be independent of the bacterial host. We also demonstrated that the Tp mutation results in a lower expression of the *traJ* gene. TraJ has been demonstrated as an essential conjugative protein that negatively autoregulates the expression of the relaxase operon [64]. Therefore, decreased expression of *traJ* could have a significant effect on the expression of all the conjugative machinery proteins. Further investigation will focus on resolving the link between *traJ* downregulation and increased yeast viability or DNA transfer during the co-culture conjugation step.

The significantly improved pSC5 plasmid allowed for DNA transfer to seven *Metschnikowia* and *Candida* yeast species, though relatively few colonies were obtained for each of them. One explanation for the low conjugative transfer could be that the *S. cerevisiae* centromere along with the origin of replication was not functional in these yeasts. In such a case, survival of these yeasts would only be possible if the conjugative plasmid was integrated into the yeast genome. Using a plasmid rescue experiment, we showed that plasmids could be recovered in *E. coli*, although none of them had the correct size or ability to conjugate. Since *E. coli* can assemble linear fragments into plasmids [65], it is most likely that some of the linear yeast fragments with integrated conjugative plasmids were assembled into plasmids in *E. coli*. Despite not being able to replicate as an episome in diverse yeasts, the improved conjugative plasmids, especially pSC5GGv1_ShBle with two antibiotic resistance genes, provide a great initial resource for DNA delivery. For applications where replicative plasmids are necessary for the yeast species of interest, specific origins and centromeres will need to be identified and incorporated into the conjugative plasmid as was done for *S. cerevisiae* [66, 67].

Our improved conjugative plasmids hold promise as a novel antifungal. As a proof of concept, we cloned restriction nucleases onto pSC5GGv2 plasmid and demonstrated that >99% of yeast cells that receive the plasmid DNA can be eliminated. However, additional improvements in the conjugation frequency will need to be achieved before this technology can be used in antifungal treatments. In the future, our Golden Gate-compatible plasmids can be engineered with programmable systems such as CRISPR/Cas9 to target specific yeast strains. This coincides with the development and optimization of numerous CRISPR-based editing platforms optimized for a diversity of yeast species [68–72], including *Candida* pathogens [73]. Recent work has demonstrated the utility of CRISPR systems for modifying fungal genes involved in virulence [74–81] and antifungal drug resistance [74, 77, 82, 83] in diverse *Candida* pathogens, and combining these CRISPR systems with this trans-kingdom conjugation system could facilitate the delivery of CRISPR to fungi in different environmental contexts.

## Data Availability

Raw data used to support the findings presented in this study are available from the corresponding author upon request.

## Supplementary Materials

Supplementary Materials Supplemental Figure S1. Deletion plasmid assembly strategy. Supplemental Figure S2. Bacterial conjugation from S. meliloti to S. cerevisiae. Supplemental Figure S3. Quantitative real-time polymerase chain reaction (qRT-PCR) of traJ expression. Supplemental Figure S4. Bacterial conjugation from E. coli to diverse yeast species. Supplemental Figure S5. Conjugation frequency of pSC5 to Metschnikowia gruessi. Supplemental Figure S6. Genotyping transconjugants of diverse yeast species. Supplemental Figure S7. Phenotypic E. coli to E. coli conjugation screen of recovered transconjugant plasmids from diverse yeast species. Supplemental Table S1. Description of pTA-Mob 2.0 deletion plasmid library. Supplemental Table S2. List of primers used to amplify the assembly fragments and genotype the plasmids created in this study. Supplemental Table S3. Conjugation phenotype of pTA-Mob 2.0 deletion plasmid library. Supplemental Table S4. Whole plasmid sequencing of minimal conjugative plasmid 3 (M3C1 and M3C2). Supplemental Table S5. Cis- and trans- conjugation of super conjugative plasmid (pSC5). Supplemental Table S6. Recipient yeast cell concentrations used in conjugation experiments of Figure 4. Supplemental Table S7. S. cerevisiae cell viability following conjugation with different E. coli strains. Supplemental Table S8. S. cerevisiae cell viability following conjugation with different S. meliloti strains Supplemental Table S9. S. cerevisiae transconjugant colony count following conjugation with S. meliloti. Supplemental Table S10. Yeast transconjugant colony counts for the conjugation-based antifungal experiment (Figure 7).

## References

[1] M. C. Fisher, S. J. Gurr, C. A. Cuomo, D. S. Blehert, H. Jin, E. H. Stukenbrock, J. E. Stajich, R. Kahmann, C. Boone, D. W. Denning, N. A. R. Gow, B. S. Klein, J. W. Kronstad, D. C. Sheppard, J. W. Taylor, G. D. Wright, J. Heitman, A. Casadevall, and L. E. Cowen, “Threats posed by the fungal kingdom to humans, wildlife, and agriculture,” *MBio*, vol. 11, no. 3, article e00449, 2020 10.1128/mBio.00449-20 PMC7403777 32371596

[2] K. D. Hyde, J. Xu, S. Rapior, R. Jeewon, S. Lumyong, A. G. T. Niego, P. D. Abeywickrama, J. V. S. Aluthmuhandiram, R. S. Brahamanage, S. Brooks, A. Chaiyasen, K. W. T. Chethana, P. Chomnunti, C. Chepkirui, B. Chuankid, N. I. de Silva, M. Doilom, C. Faulds, E. Gentekaki, V. Gopalan, P. Kakumyan, D. Harishchandra, H. Hemachandran, S. Hongsanan, A. Karunarathna, S. C. Karunarathna, S. Khan, J. Kumla, R. S. Jayawardena, J.-K. Liu, N. Liu, T. Luangharn, A. P. G. Macabeo, D. S. Marasinghe, D. Meeks, P. E. Mortimer, P. Mueller, S. Nadir, K. N. Nataraja, S. Nontachaiyapoom, M. O’Brien, W. Penkhrue, C. Phukhamsakda, U. S. Ramanan, A. R. Rathnayaka, R. B. Sadaba, B. Sandargo, B. C. Samarakoon, D. S. Tennakoon, R. Siva, W. Sriprom, T. S. Suryanarayanan, K. Sujarit, N. Suwannarach, T. Suwunwong, B. Thongbai, N. Thongklang, D. Wei, S. N. Wijesinghe, J. Winiski, J. Yan, E. Yasanthika, and M. Stadler, “The amazing potential of fungi: 50 ways we can exploit fungi industrially,” *Fungal Diversity*, vol. 97, no. 1, pp. 1–136, 2019

[3] M. Parapouli, A. Vasileiadis, A. S. Afendra, and E. Hatziloukas, “Saccharomyces cerevisiae and its industrial applications,” *AIMS microbiology*, vol. 6, no. 1, pp. 1–32, 2020 32226912 10.3934/microbiol.2020001 PMC7099199

[4] L. Edwards-Ingram, P. Gitsham, N. Burton, G. Warhurst, I. Clarke, D. Hoyle, S. G. Oliver, and L. Stateva, “Genotypic and physiological characterization of Saccharomyces boulardii, the probiotic strain of Saccharomyces cerevisiae,” *Applied and Environmental Microbiology*, vol. 73, no. 8, pp. 2458–2467, 2007 17293506 10.1128/AEM.02201-06 PMC1855594

[5] A. Everard, S. Matamoros, L. Geurts, N. M. Delzenne, and P. D. Cani, “Saccharomyces boulardii administration changes gut microbiota and reduces hepatic steatosis, low-grade inflammation, and fat mass in obese and type 2 diabetic db/db mice,” *MBio*, vol. 5, no. 3, pp. e01011–e01014, 2014 24917595 10.1128/mBio.01011-14 PMC4056549

[6] P. G. Pappas, M. S. Lionakis, M. C. Arendrup, L. Ostrosky-Zeichner, and B. J. Kullberg, “Invasive candidiasis,” *Nature Reviews Disease Primers*, vol. 4, no. 1, pp. 1–20, 2018 10.1038/nrdp.2018.26 29749387

[7] J. J. Limon, J. Tang, D. Li, A. J. Wolf, K. S. Michelsen, V. Funari, M. Gargus, C. Nguyen, P. Sharma, V. I. Maymi, I. D. Iliev, J. H. Skalski, J. Brown, C. Landers, J. Borneman, J. Braun, S. R. Targan, D. P. B. McGovern, and D. M. Underhill, “*Malassezia* is associated with Crohn’s disease and exacerbates colitis in mouse models,” *Cell Host & Microbe*, vol. 25, no. 3, pp. 377–388.e6, 2019 30850233 10.1016/j.chom.2019.01.007 PMC6417942

[8] B. Aykut, S. Pushalkar, R. Chen, Q. Li, R. Abengozar, J. I. Kim, S. A. Shadaloey, D. Wu, P. Preiss, N. Verma, Y. Guo, A. Saxena, M. Vardhan, B. Diskin, W. Wang, J. Leinwand, E. Kurz, J. A. Kochen Rossi, M. Hundeyin, C. Zambrinis, X. Li, D. Saxena, and G. Miller, “The fungal mycobiome promotes pancreatic oncogenesis via activation of MBL,” *Nature*, vol. 574, no. 7777, pp. 264–267, 2019 31578522 10.1038/s41586-019-1608-2 PMC6858566

[9] D. M. Proctor, T. Dangana, D. J. Sexton, C. Fukuda, R. D. Yelin, M. Stanley, P. B. Bell, S. Baskaran, C. Deming, Q. Chen, S. Conlan, M. Park, N. I. S. C. Comparative Sequencing Program, R. M. Welsh, S. Vallabhaneni, T. Chiller, K. Forsberg, S. R. Black, M. Pacilli, H. H. Kong, M. Y. Lin, M. E. Schoeny, A. P. Litvintseva, J. A. Segre, and M. K. Hayden, “Integrated genomic, epidemiologic investigation of *Candida auris* skin colonization in a skilled nursing facility,” *Nature Medicine*, vol. 27, no. 8, pp. 1401–1409, 2021 10.1038/s41591-021-01383-w PMC9396956 34155414

[10] H. Du, J. Bing, T. Hu, C. L. Ennis, C. J. Nobile, and G. Huang, “Candida auris: epidemiology, biology, antifungal resistance, and virulence,” *PLoS Pathogens*, vol. 16, no. 10, article e1008921, 2020 10.1371/journal.ppat.1008921 PMC7581363 33091071

[11] J. Geddes-McAlister, and R. S. Shapiro, “New pathogens, new tricks: emerging, drug-resistant fungal pathogens and future prospects for antifungal therapeutics,” *Annals of the New York Academy of Sciences*, vol. 1435, no. 1, pp. 57–78, 2019 29762860 10.1111/nyas.13739

[12] D. Palacios Araya, K. L. Palmer, and B. A. Duerkop, “CRISPR-based antimicrobials to obstruct antibiotic-resistant and pathogenic bacteria,” *PLoS Pathogens*, vol. 17, no. 7, article e1009672, 2021 10.1371/journal.ppat.1009672 PMC8266055 34237097

[13] R. J. Citorik, M. Mimee, and T. K. Lu, “Sequence-specific antimicrobials using efficiently delivered RNA-guided nucleases,” *Nature Biotechnology*, vol. 32, no. 11, pp. 1141–1145, 2014 10.1038/nbt.3011 PMC4237163 25240928

[14] K. Kiga, X. E. Tan, R. Ibarra-Chávez, S. Watanabe, Y. Aiba, Y. Sato’o, F. Y. Li, T. Sasahara, B. Cui, M. Kawauchi, T. Boonsiri, K. Thitiananpakorn, Y. Taki, A. H. Azam, M. Suzuki, J. R. Penadés, and L. Cui, “Development of CRISPR-Cas13a-based antimicrobials capable of sequence- specific killing of target bacteria,” *Nature Communications*, vol. 11, no. 1, p. 2934, 2020 10.1038/s41467-020-16731-6 PMC7287087 32523110

[15] A. Chavez, B. W. Pruitt, M. Tuttle, R. S. Shapiro, R. J. Cecchi, J. Winston, B. M. Turczyk, M. Tung, J. J. Collins, and G. M. Church, “Precise Cas9 targeting enables genomic mutation prevention,” *Proceedings of the National Academy of Sciences of the United States of America*, vol. 115, no. 14, pp. 3669–3673, 2018 29555762 10.1073/pnas.1718148115 PMC5889643

[16] A. A. Gomaa, H. E. Klumpe, M. L. Luo, K. Selle, R. Barrangou, and C. L. Beisel, “Programmable removal of bacterial strains by use of genome-targeting CRISPR-Cas systems,” *MBio*, vol. 5, no. 1, article e00928, 2014 10.1128/mBio.00928-13 PMC3903277 24473129

[17] K. Selle, J. R. Fletcher, H. Tuson, D. S. Schmitt, L. McMillan, G. S. Vridhambal, A. J. Rivera, S. A. Montgomery, L.-C. Fortier, R. Barrangou, C. M. Theriot, and D. G. Ousterout, “In vivo targeting of Clostridioides difficile using phage-delivered CRISPR-Cas3 antimicrobials,” *MBio*, vol. 11, no. 2, pp. e00019–e00020, 2020 32156803 10.1128/mBio.00019-20 PMC7064742

[18] D. Bikard, C. W. Euler, W. Jiang, P. M. Nussenzweig, G. W. Goldberg, X. Duportet, V. A. Fischetti, and L. A. Marraffini, “Exploiting CRISPR-Cas nucleases to produce sequence-specific antimicrobials,” *Nature Biotechnology*, vol. 32, no. 11, pp. 1146–1150, 2014 10.1038/nbt.3043 PMC4317352 25282355

[19] T. A. Hamilton, G. M. Pellegrino, J. A. Therrien, D. T. Ham, P. C. Bartlett, B. J. Karas, G. B. Gloor, and D. R. Edgell, “Efficient inter-species conjugative transfer of a CRISPR nuclease for targeted bacterial killing,” *Nature Communications*, vol. 10, no. 1, p. 4544, 2019 10.1038/s41467-019-12448-3 PMC6778077 31586051

[20] R. López-Igual, J. Bernal-Bayard, A. Rodríguez-Patón, J.-M. Ghigo, and D. Mazel, “Engineered toxin-intein antimicrobials can selectively target and kill antibiotic-resistant bacteria in mixed populations,” *Nature Biotechnology*, vol. 37, no. 7, pp. 755–760, 2019 10.1038/s41587-019-0105-3 30988505

[21] K. Neil, N. Allard, P. Roy, F. Grenier, A. Menendez, V. Burrus, and S. Rodrigue, “High-efficiency delivery of CRISPR-Cas9 by engineered probiotics enables precise microbiome editing,” *Molecular Systems Biology*, vol. 17, no. 10, article e10335, 2021 10.15252/msb.202110335 PMC8527022 34665940

[22] K. Neil, N. Allard, F. Grenier, V. Burrus, and S. Rodrigue, “Highly efficient gene transfer in the mouse gut microbiota is enabled by the Incl_2_ conjugative plasmid TP114,” *Communications biology*, vol. 3, no. 1, pp. 1–9, 2020 32963323 10.1038/s42003-020-01253-0 PMC7508951

[23] M. Rodrigues, S. W. McBride, K. Hullahalli, K. L. Palmer, and B. A. Duerkop, “Conjugative delivery of CRISPR-Cas9 for the selective depletion of antibiotic-resistant enterococci,” *Antimicrobial Agents and Chemotherapy*, vol. 63, no. 11, article e01454, 2019 10.1128/AAC.01454-19 PMC6811441 31527030

[24] B. J. Karas, R. E. Diner, S. C. Lefebvre, J. McQuaid, A. P. R. Phillips, C. M. Noddings, J. K. Brunson, R. E. Valas, T. J. Deerinck, J. Jablanovic, J. T. F. Gillard, K. Beeri, M. H. Ellisman, J. I. Glass, C. A. Hutchison, H. O. Smith, J. C. Venter, A. E. Allen, C. L. Dupont, and P. D. Weyman, “Designer diatom episomes delivered by bacterial conjugation,” *Nature Communications*, vol. 6, no. 1, p. 6925, 2015 10.1038/ncomms7925 PMC4411287 25897682

[25] S. L. Brumwell, M. R. Mac Leod, T. Huang, R. Cochrane, R. S. Meaney, M. Zamani, O. Matysiakiewicz, K. N. Dan, P. Janakirama, D. R. Edgell, T. C. Charles, T. M. Finan, and B. J. Karas, “Designer Sinorhizobium meliloti strains and multi-functional vectors enable direct inter-kingdom DNA transfer,” *PLoS One*, vol. 14, no. 6, article e0206781, 2019 10.1371/journal.pone.0206781 PMC6576745 31206509

[26] G. T. Hayman, and P. L. Bolen, “Movement of shuttle plasmids from *Escherichia coli* into yeasts other than *Saccharomyces cerevisiae* using trans-kingdom conjugation,” *Plasmid*, vol. 30, no. 3, pp. 251–257, 1993 8302932 10.1006/plas.1993.1056

[27] K. Moriguchi, N. Edahiro, S. Yamamoto, K. Tanaka, N. Kurata, and K. Suzuki, “Transkingdom genetic transfer from Escherichia coli to Saccharomyces cerevisiae as a simple gene introduction tool,” *Applied and Environmental Microbiology*, vol. 79, no. 14, pp. 4393–4400, 2013 23666333 10.1128/AEM.00770-13 PMC3697487

[28] M. P. M. Soltysiak, R. S. Meaney, S. Hamadache, P. Janakirama, D. R. Edgell, and B. J. Karas, “Trans-kingdom conjugation within solid media from Escherichia coli to Saccharomyces cerevisiae,” *International Journal of Molecular Sciences*, vol. 20, no. 20, p. 5212, 2019 31640164 10.3390/ijms20205212 PMC6829330

[29] F. I. R. M. Zoolkefli, K. Moriguchi, Y. Cho, K. Kiyokawa, S. Yamamoto, and K. Suzuki, “Isolation and analysis of donor chromosomal genes whose deficiency is responsible for accelerating bacterial and trans-kingdom conjugations by IncP1 T4SS machinery,” *Frontiers in Microbiology*, vol. 12, p. 971, 2021 10.3389/fmicb.2021.620535 PMC8174662 34093458

[30] P. Norberg, M. Bergström, V. Jethava, D. Dubhashi, and M. Hermansson, “The IncP-1 plasmid backbone adapts to different host bacterial species and evolves through homologous recombination,” *Nature Communications*, vol. 2, no. 1, p. 268, 2011 10.1038/ncomms1267 PMC3104523 21468020

[31] W. Pansegrau, E. Lanka, P. T. Barth, D. H. Figurski, D. G. Guiney, D. Haas, D. R. Helinski, H. Schwab, V. A. Stanisich, and C. M. Thomas, “Complete nucleotide sequence of Birmingham IncP*α* plasmids: compilation and comparative analysis,” *Journal of Molecular Biology*, vol. 239, no. 5, pp. 623–663, 1994 8014987 10.1006/jmbi.1994.1404

[32] G. Ziegelin, J. P. Fürste, and E. Lanka, “TraJ protein of plasmid RP4 binds to a 19-base pair invert sequence repetition within the transfer origin,” *The Journal of Biological Chemistry*, vol. 264, no. 20, pp. 11989–11994, 1989 2663846

[33] J. P. Fürste, W. Pansegrau, G. Ziegelin, M. Kröger, and E. Lanka, “Conjugative transfer of promiscuous IncP plasmids: interaction of plasmid-encoded products with the transfer origin,” *Proceedings of the National Academy of Sciences of the United States of America*, vol. 86, no. 6, pp. 1771–1775, 1989 2538813 10.1073/pnas.86.6.1771 PMC286786

[34] W. Pansegrau, D. Balzer, V. Kruft, R. Lurz, and E. Lanka, “In vitro assembly of relaxosomes at the transfer origin of plasmid RP4,” *Proceedings of the National Academy of Sciences of the United States of America*, vol. 87, no. 17, pp. 6555–6559, 1990 2168553 10.1073/pnas.87.17.6555 PMC54575

[35] W. Pansegrau, W. Schröder, and E. Lanka, “Relaxase (TraI) of IncP alpha plasmid RP4 catalyzes a site-specific cleaving-joining reaction of single-stranded DNA,” *Proceedings of the National Academy of Sciences of the United States of America*, vol. 90, no. 7, pp. 2925–2929, 1993 8385350 10.1073/pnas.90.7.2925 PMC46209

[36] W. Pansegrau, and E. Lanka, “Mechanisms of initiation and termination reactions in conjugative DNA processing:,” *The Journal of Biological Chemistry*, vol. 271, no. 22, pp. 13068–13076, 1996 8662726 10.1074/jbc.271.22.13068

[37] G. Ziegelin, W. Pansegrau, R. Lurz, and E. Lanka, “TraK protein of conjugative plasmid RP4 forms a specialized nucleoprotein complex with the transfer origin,” *The Journal of Biological Chemistry*, vol. 267, no. 24, pp. 17279–17286, 1992 1324929

[38] C. E. Rees, and B. M. Wilkins, “Protein transfer into the recipient cell during bacterial conjugation: studies with F and RP4,” *Molecular Microbiology*, vol. 4, no. 7, pp. 1199–1205, 1990 2172695 10.1111/j.1365-2958.1990.tb00695.x

[39] E. Cabezón, J. I. Sastre, and F. de la Cruz, “Genetic evidence of a coupling role for the TraG protein family in bacterial conjugation,” *Molecular & General Genetics*, vol. 254, no. 4, pp. 400–406, 1997 9180693 10.1007/s004380050432

[40] A. M. Grahn, J. Haase, D. H. Bamford, and E. Lanka, “Components of the RP4 conjugative transfer apparatus form an envelope structure bridging inner and outer membranes of donor cells: implications for related macromolecule transport systems,” *Journal of Bacteriology*, vol. 182, no. 6, pp. 1564–1574, 2000 10692361 10.1128/jb.182.6.1564-1574.2000 PMC94453

[41] R. Eisenbrandt, M. Kalkum, R. Lurz, and E. Lanka, “Maturation of IncP pilin precursors resembles the catalytic dyad-like mechanism of leader peptidases,” *Journal of Bacteriology*, vol. 182, no. 23, pp. 6751–6761, 2000 11073921 10.1128/jb.182.23.6751-6761.2000 PMC111419

[42] R. Eisenbrandt, M. Kalkum, E. M. Lai, R. Lurz, C. I. Kado, and E. Lanka, “Conjugative pili of IncP plasmids, and the Ti plasmid T pilus are composed of cyclic subunits,” *The Journal of Biological Chemistry*, vol. 274, no. 32, pp. 22548–22555, 1999 10428832 10.1074/jbc.274.32.22548

[43] R. R. Cochrane, S. L. Brumwell, A. Shrestha, D. J. Giguere, S. Hamadache, G. B. Gloor, D. R. Edgell, and B. J. Karas, “Cloning of Thalassiosira pseudonana’s mitochondrial genome in Saccharomyces cerevisiae and Escherichia coli,” *Biology*, vol. 9, no. 11, p. 358, 2020 33114477 10.3390/biology9110358 PMC7693118

[44] B. J. Karas, J. Jablanovic, E. Irvine, L. Sun, L. Ma, P. D. Weyman, D. G. Gibson, J. I. Glass, J. C. Venter, C. A. Hutchison, H. O. Smith, and Y. Suzuki, “Transferring whole genomes from bacteria to yeast spheroplasts using entire bacterial cells to reduce DNA shearing,” *Nature Protocols*, vol. 9, no. 4, pp. 743–750, 2014 24603933 10.1038/nprot.2014.045

[45] R. R. Cochrane, S. L. Brumwell, M. P. M. Soltysiak, S. Hamadache, J. G. Davis, J. Wang, S. Q. Tholl, P. Janakirama, D. R. Edgell, and B. J. Karas, “Rapid method for generating designer algal mitochondrial genomes,” *Algal Research*, vol. 50, article 102014, 2020

[46] Z. B. Gordon, M. P. M. Soltysiak, C. Leichthammer, J. A. Therrien, R. S. Meaney, C. Lauzon, M. Adams, D. K. Lee, P. Janakirama, M.-A. Lachance, and B. J. Karas, “Development of a transformation method for Metschnikowia borealis and other CUG-Serine Yeasts,” *Genes*, vol. 10, no. 2, p. 78, 2019 30678093 10.3390/genes10020078 PMC6409616

[47] B. J. Karas, C. Tagwerker, I. T. Yonemoto, C. A. Hutchison, and H. O. Smith, “Cloning the Acholeplasma laidlawii PG-8A genome in Saccharomyces cerevisiae as a yeast centromeric plasmid,” *ACS Synthetic Biology*, vol. 1, no. 1, pp. 22–28, 2012 23651007 10.1021/sb200013j

[48] S. Marillonnet, and R. Grützner, “Synthetic DNA assembly using golden gate cloning and the hierarchical modular cloning pipeline,” *Current Protocols in Molecular Biology*, vol. 130, no. 1, article e115, 2020 10.1002/cpmb.115 32159931

[49] C. Engler, R. Kandzia, and S. Marillonnet, “A one pot, one step, precision cloning method with high throughput capability,” *PLoS One*, vol. 3, no. 11, article e3647, 2008 10.1371/journal.pone.0003647 PMC2574415 18985154

[50] R. Ng, and J. Abelson, “Isolation and sequence of the gene for actin in Saccharomyces cerevisiae,” *Proceedings of the National Academy of Sciences of the United States of America*, vol. 77, no. 7, pp. 3912–3916, 1980 7001447 10.1073/pnas.77.7.3912 PMC349737

[51] P. Ruotsalainen, R. Penttinen, S. Mattila, and M. Jalasvuori, “Midbiotics: conjugative plasmids for genetic engineering of natural gut flora,” *Gut Microbes*, vol. 10, no. 6, pp. 643–653, 2019 30951393 10.1080/19490976.2019.1591136 PMC6866695

[52] K. Hullahalli, M. Rodrigues, and K. L. Palmer, “Exploiting CRISPR-Cas to manipulate Enterococcus faecalis populations,” *eLife*, vol. 6, article e26664, 2017 10.7554/eLife.26664 PMC5491264 28644125

[53] G. Ram, H. F. Ross, R. P. Novick, I. Rodriguez-Pagan, and D. Jiang, “Conversion of staphylococcal pathogenicity islands to CRISPR-carrying antibacterial agents that cure infections in mice,” *Nature Biotechnology*, vol. 36, no. 10, pp. 971–976, 2018 10.1038/nbt.4203 PMC6511514 30247487

[54] K. N. Lam, P. Spanogiannopoulos, P. Soto-Perez, M. Alexander, M. J. Nalley, J. E. Bisanz, R. R. Nayak, A. M. Weakley, F. B. Yu, and P. J. Turnbaugh, “Phage-delivered CRISPR-Cas9 for strain-specific depletion and genomic deletions in the gut microbiome,” *Cell Reports*, vol. 37, no. 5, article 109930, 2021 10.1016/j.celrep.2021.109930 PMC8591988 34731631

[55] R. B. Vercoe, J. T. Chang, R. L. Dy, C. Taylor, T. Gristwood, J. S. Clulow, C. Richter, R. Przybilski, A. R. Pitman, and P. C. Fineran, “Cytotoxic chromosomal targeting by CRISPR/Cas systems can reshape bacterial genomes and expel or remodel pathogenicity islands,” *PLoS Genetics*, vol. 9, no. 4, article e1003454, 2013 10.1371/journal.pgen.1003454 PMC3630108 23637624

[56] J.-S. Kim, D.-H. Cho, M. Park, W.-J. Chung, D. Shin, K. S. Ko, and D.-H. Kweon, “CRISPR/Cas9-mediated re-sensitization of antibiotic-resistant Escherichia coli harboring extended-spectrum *β*-lactamases,” *Journal of Microbiology and Biotechnology*, vol. 26, no. 2, pp. 394–401, 2016 26502735 10.4014/jmb.1508.08080

[57] P. Wang, D. He, B. Li, Y. Guo, W. Wang, X. Luo, X. Zhao, and X. Wang, “Eliminating mcr-1-harbouring plasmids in clinical isolates using the CRISPR/Cas9 system,” *The Journal of Antimicrobial Chemotherapy*, vol. 74, no. 9, pp. 2559–2565, 2019 31203365 10.1093/jac/dkz246

[58] J. A. Valderrama, J. Andrés Valderrama, S. S. Kulkarni, V. Nizet, and E. Bier, “A bacterial gene-drive system efficiently edits and inactivates a high copy number antibiotic resistance locus,” *Nature Communications*, vol. 10, no. 1, pp. 1–8, 2019 10.1038/s41467-019-13649-6 PMC6915771 31844051

[59] K. Wang, and M. Nicholaou, “Suppression of antimicrobial resistance in MRSA using CRISPR-dCas9,” *Clinical Laboratory Science*, vol. 30, no. 4, pp. 207–213, 2017

[60] Q. Li, P. Zhao, L. Li, H. Zhao, L. Shi, and P. Tian, “Engineering a CRISPR interference system to repress a class 1 integron in Escherichia coli,” *Antimicrobial Agents and Chemotherapy*, vol. 64, no. 3, article e01789, 2020 10.1128/AAC.01789-19 PMC7038292 31871091

[61] J. Y. Park, B. Y. Moon, J. W. Park, J. A. Thornton, Y. H. Park, and K. S. Seo, “Genetic engineering of a temperate phage-based delivery system for CRISPR/Cas9 antimicrobials against *Staphylococcus aureus*,” *Scientific Reports*, vol. 7, no. 1, p. 44929, 2017 28322317 10.1038/srep44929 PMC5359561

[62] R. D. Gietz, and R. A. Woods, “Genetic transformation of yeast,” *Biotechniques*, vol. 30, no. 4, pp. 816–831, 2001 11314265 10.2144/01304rv02

[63] J. F. Martín, “Fungal transformation: from protoplasts to targeted recombination systems,” *Genetic Transformation Systems in Fungi*, M. A. van den Berg, and K. Maruthachalam, Eds., Springer International Publishing, Cham, vol. 1, pp. 3–18, 2015

[64] M. Zatyka, G. Jagura-Burdzy, and C. M. Thomas, “Regulation of transfer genes of promiscuous IncP alpha plasmid RK2: repression of Tra1 region transcription both by relaxosome proteins and by the Tra2 regulator TrbA,” *Microbiology*, vol. 140, no. 11, pp. 2981–2990, 1994 7812437 10.1099/13500872-140-11-2981

[65] M. Kostylev, A. E. Otwell, R. E. Richardson, and Y. Suzuki, “Cloning should be simple: Escherichia coli DH5*α*-mediated assembly of multiple DNA fragments with short end homologies,” *PLoS One*, vol. 10, no. 9, article e0137466, 2015 10.1371/journal.pone.0137466 PMC4562628 26348330

[66] D. T. Stinchcomb, K. Struhl, and R. W. Davis, “Isolation and characterisation of a yeast chromosomal replicator,” *Nature*, vol. 282, no. 5734, pp. 39–43, 1979 388229 10.1038/282039a0

[67] L. Clarke, and J. Carbon, “Isolation of a yeast centromere and construction of functional small circular chromosomes,” *Nature*, vol. 287, no. 5782, pp. 504–509, 1980 6999364 10.1038/287504a0

[68] P. Cai, J. Gao, and Y. Zhou, “CRISPR-mediated genome editing in non-conventional yeasts for biotechnological applications,” *Microbial Cell Factories*, vol. 18, no. 1, p. 63, 2019 30940138 10.1186/s12934-019-1112-2 PMC6444819

[69] F. Morio, L. Lombardi, and G. Butler, “The CRISPR toolbox in medical mycology: state of the art and perspectives,” *PLoS Pathogens*, vol. 16, no. 1, article e1008201, 2020 10.1371/journal.ppat.1008201 PMC6964833 31945142

[70] H. Raschmanová, A. Weninger, A. Glieder, K. Kovar, and T. Vogl, “Implementing CRISPR-Cas technologies in conventional and non-conventional yeasts: current state and future prospects,” *Biotechnology Advances*, vol. 36, no. 3, pp. 641–665, 2018 29331410 10.1016/j.biotechadv.2018.01.006

[71] V. Stovicek, C. Holkenbrink, and I. Borodina, “CRISPR/Cas system for yeast genome engineering: advances and applications,” *FEMS Yeast Research*, vol. 17, no. 5, 2017 10.1093/femsyr/fox030 PMC5812514 28505256

[72] L. Shan, Z. Dai, and Q. Wang, “Advances and opportunities of CRISPR/Cas technology in bioengineering non-conventional yeasts,” *Frontiers in Bioengineering and Biotechnology*, vol. 9, article 765396, 2021 10.3389/fbioe.2021.765396 PMC8542773 34708030

[73] D. Uthayakumar, J. Sharma, L. Wensing, and R. S. Shapiro, “CRISPR-based genetic manipulation of *Candida* species: historical perspectives and current approaches,” *Frontiers in Genome Editing*, vol. 2, article 606281, 2021 10.3389/fgeed.2020.606281 PMC8525362 34713231

[74] V. K. Vyas, M. I. Barrasa, and G. R. Fink, “A *Candida albicans* CRISPR system permits genetic engineering of essential genes and gene families,” *Science Advances*, vol. 1, no. 3, article e1500248, 2015 10.1126/sciadv.1500248 PMC4428347 25977940

[75] L. Enkler, D. Richer, A. L. Marchand, D. Ferrandon, and F. Jossinet, “Genome engineering in the yeast pathogen *Candida glabrata* using the CRISPR-Cas9 system,” *Scientific Reports*, vol. 6, no. 1, p. 35766, 2016 27767081 10.1038/srep35766 PMC5073330

[76] S. Rosiana, L. Zhang, G. H. Kim, A. V. Revtovich, D. Uthayakumar, A. Sukumaran, J. Geddes-McAlister, N. V. Kirienko, and R. S. Shapiro, “Comprehensive genetic analysis of adhesin proteins and their role in virulence of *Candida albicans*,” *Genetics*, vol. 217, no. 2, 2021 10.1093/genetics/iyab003 PMC8045720 33724419

[77] R. S. Shapiro, A. Chavez, C. B. M. Porter, M. Hamblin, C. S. Kaas, J. E. DiCarlo, G. Zeng, X. Xu, A. V. Revtovich, N. V. Kirienko, Y. Wang, G. M. Church, and J. J. Collins, “A CRISPR-Cas9-based gene drive platform for genetic interaction analysis in *Candida albicans*,” *Nature Microbiology*, vol. 3, no. 1, pp. 73–82, 2018 10.1038/s41564-017-0043-0 PMC5832965 29062088

[78] M. Zoppo, M. D. Luca, S. N. Villarreal, N. Poma, M. I. Barrasa, D. Bottai, V. K. Vyas, and A. Tavanti, “A CRISPR/Cas9-based strategy to simultaneously inactivate the entire ALS gene family in Candida orthopsilosis,” *Future Microbiology*, vol. 14, no. 16, pp. 1383–1396, 2019 31659913 10.2217/fmb-2019-0168

[79] D. J. Santana, and T. R. O’Meara, “Forward and reverse genetic dissection of morphogenesis identifies filament- competent *Candida auris* strains,” *Nature Communications*, vol. 12, no. 1, pp. 1–13, 2021 10.1038/s41467-021-27545-5 PMC8664941 34893621

[80] M. Y. Huang, C. A. Woolford, G. May, C. J. McManus, and A. P. Mitchell, “Circuit diversification in a biofilm regulatory network,” *PLoS Pathogens*, vol. 15, no. 5, article e1007787, 2019 10.1371/journal.ppat.1007787 PMC6530872 31116789

[81] K. Min, A. Biermann, D. A. Hogan, and J. B. Konopka, “Genetic analysis of *NDT80* family transcription factors in *Candida albicans* using new CRISPR-Cas9 approaches,” *Msphere*, vol. 3, no. 6, article e00545, 2018 10.1128/mSphere.00545-18 PMC6249646 30463924

[82] L. Wensing, J. Sharma, D. Uthayakumar, Y. Proteau, A. Chavez, and R. S. Shapiro, “A CRISPR interference platform for efficient genetic repression in *Candida albicans*,” *Msphere*, vol. 4, no. 1, pp. e00002–e00019, 2019 30760609 10.1128/mSphere.00002-19 PMC6374589

[83] C. L. Ennis, A. D. Hernday, and C. J. Nobile, “A markerless CRISPR-mediated system for genome editing in Candida auris reveals a conserved role for Cas5 in the caspofungin response,” *Microbiology spectrum*, vol. 9, no. 3, article e0182021, 2021 10.1128/Spectrum.01820-21 PMC8567271 34730409

