# Supplementary material for: Superior Conjugative Plasmids Delivered by Bacteria to Diverse Fungi
Source: Biodes Res. 2022 Aug 19;2022:9802168. doi: 10.34133/2022/9802168 (PMC10521675; doi:10.34133/2022/9802168)
Supplement: Supplementary Materials — Supplemental Figure S1. Deletion plasmid assembly strategy. Supplemental Figure S2. Bacterial conjugation from S. meliloti to S. cerevisiae. Supplemental Figure S3. Quantitative real-time polymerase chain reaction (qRT-PCR) of traJ expression. Supplemental Figure S4. Bacterial conjugation from E. coli to diverse yeast species. Supplemental Figure S5. Conjugation frequency of pSC5 to Metschnikowia gruessi. Supplemental Figure S6. Genotyping transconjugants of diverse yeast species. Supplemental Figure S7. Phenotypic E. coli to E. coli conjugation screen of recovered transconjugant plasmids from diverse yeast species. Supplemental Table S1. Description of pTA-Mob 2.0 deletion plasmid library. Supplemental Table S2. List of primers used to amplify the assembly fragments and genotype the plasmids created in this study. Supplemental Table S3. Conjugation phenotype of pTA-Mob 2.0 deletion plasmid library. Supplemental Table S4. Whole plasmid sequencing of minimal conjugative plasmid 3 (M3C1 and M3C2). Supplemental Table S5. Cis- and trans- conjugation of super conjugative plasmid (pSC5). Supplemental Table S6. Recipient yeast cell concentrations used in conjugation experiments of Figure 4. Supplemental Table S7. S. cerevisiae cell viability following conjugation with different E. coli strains. Supplemental Table S8. S. cerevisiae cell viability following conjugation with different S. meliloti strains Supplemental Table S9. S. cerevisiae transconjugant colony count following conjugation with S. meliloti. Supplemental Table S10. Yeast transconjugant colony counts for the conjugation-based antifungal experiment (Figure 7). [file 9802168.f1.docx]

**Title:** Superior conjugative plasmids delivered by bacteria to diverse fungi.

**Short Title:** Superior conjugative plasmids for yeast.

**Authors**

Ryan R. Cochrane^1†^, Arina Shrestha^1†^, Mariana M. Severo de Almeida^1†^, Michelle Agyare-Tabbi^2‡^, Stephanie L. Brumwell^1‡^, Samir Hamadache^1‡^, Jordyn S. Meaney^1‡^, Daniel P. Nucifora^1‡^, Henry Heng Say^1‡^, Jehoshua Sharma^2‡^, Maximillian P. M. Soltysiak^3‡^, Cheryl Tong^1‡^, Katherine Van Belois^3‡^, Emma J. L. Walker^1‡^, Marc-André Lachance^3^, Gregory B. Gloor^1^, David R. Edgell^1^, Rebecca S. Shapiro^2^, Bogumil J. Karas^1^*

^†^Co-first authors

‡Authors listed in alphabetical order

*Corresponding author. Email: [bkaras@uwo.ca](mailto:bkaras@uwo.ca)

^1^ Department of Biochemistry, Schulich School of Medicine and Dentistry, The University of Western Ontario, London, ON N6A 5C1, Canada

^2^ Department of Molecular and Cellular Biology, University of Guelph, Guelph, ON N1G 2W1, Canada

^3^ Department of Biology, The University of Western Ontario, London, Ontario, N6A 5B7, Canada

**Supplementary Figures**

**
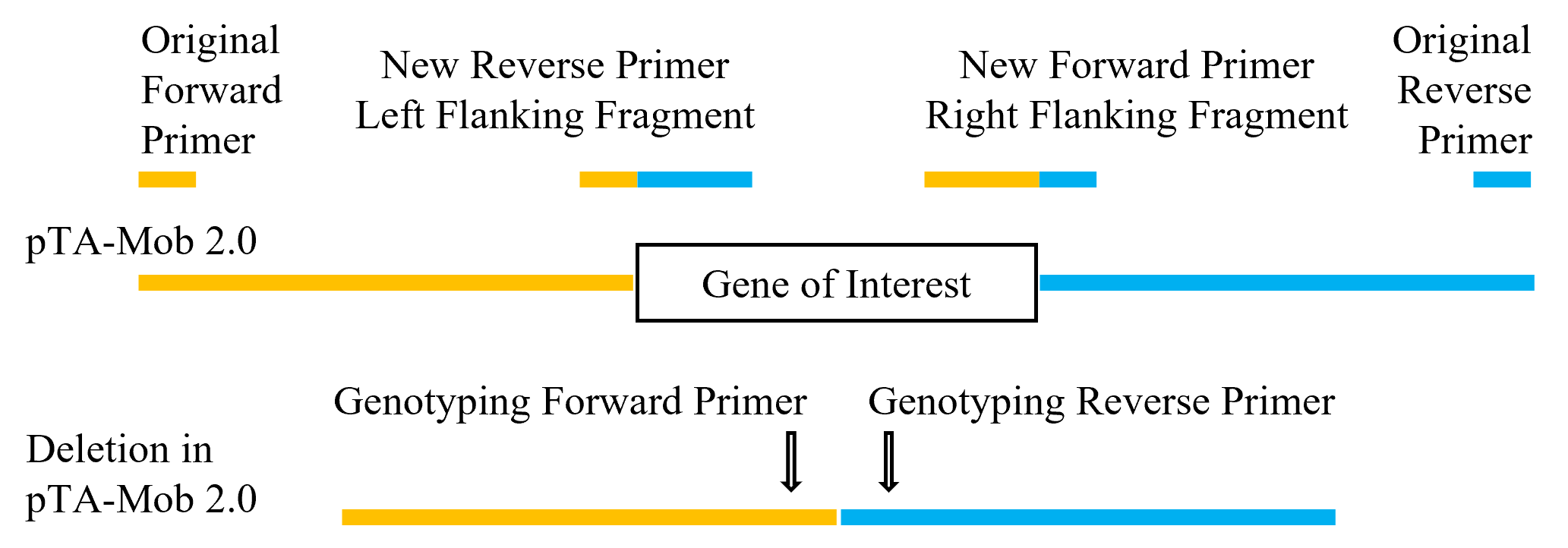
**

**Supplemental Figure S1. Deletion plasmid assembly strategy.** Each deletion plasmid was assembled from nine standard fragments as described in [(Soltysiak et al. 2019)](https://paperpile.com/c/wwpjWu/6Tuv) and two modified fragments amplified with original forward primer and new reverse primer and new forward primer and original reverse primer. After assembly, each deletion was genotyped by multiplex PCR.


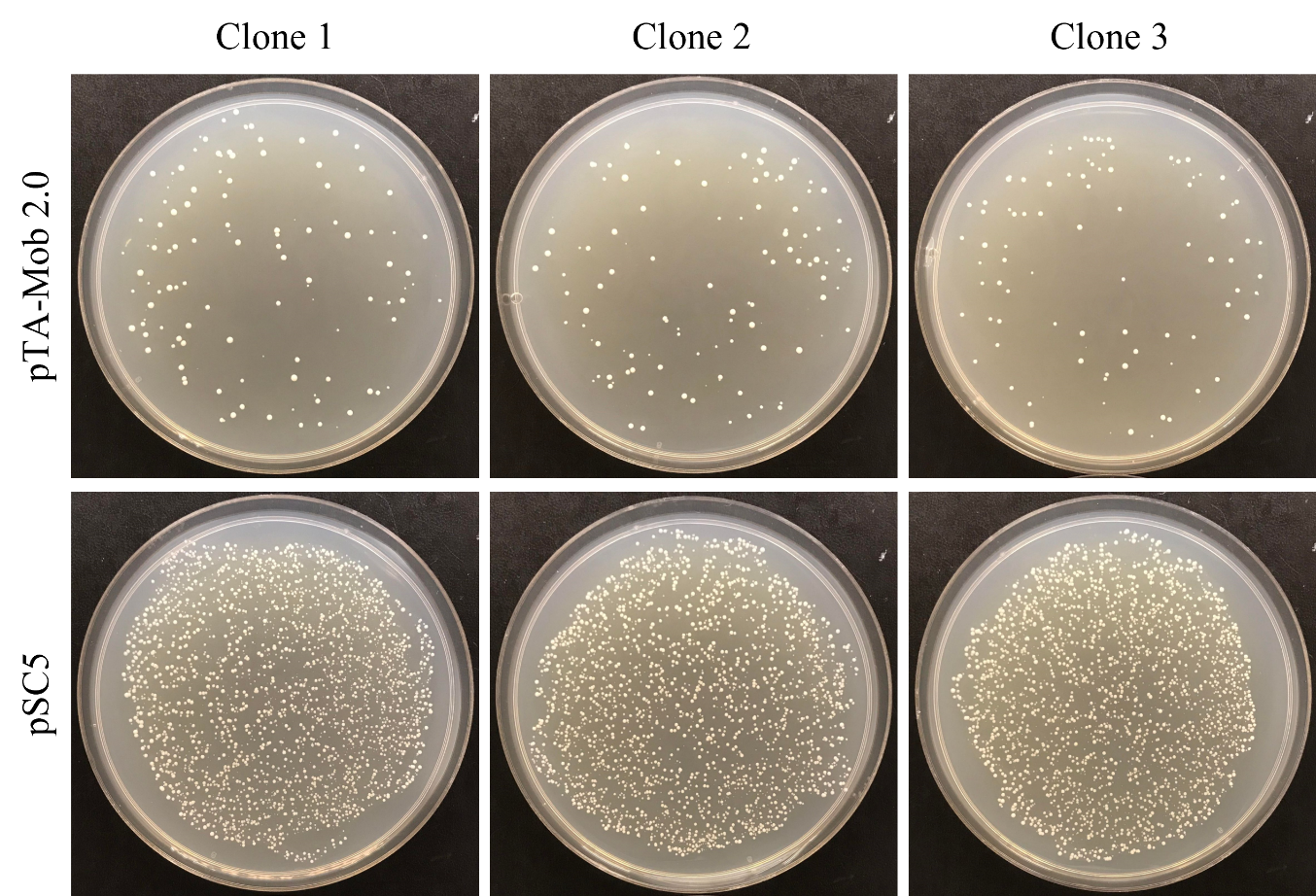


**Supplemental Figure S2. Bacterial conjugation from *S. meliloti* to *S. cerevisiae*.** Representative plates of yeast transconjugants following conjugation from three *S. meliloti* clones harboring either pTA-Mob 2.0 or pSC5, plated on minimal media lacking histidine and containing ampicillin (100 µg mL^-1^).


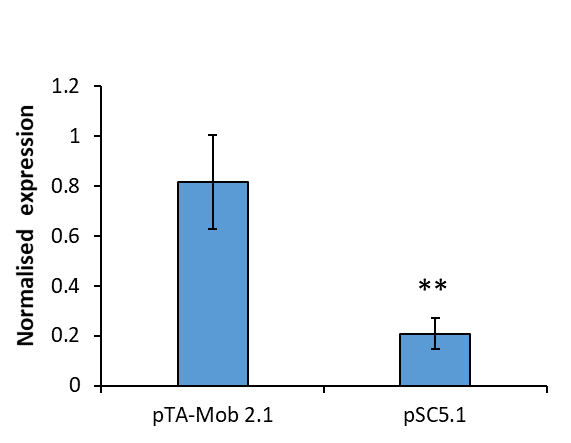


**Supplemental Figure S3. Quantitative real-time polymerase chain reaction (qRT-PCR) of *traJ* expression.** *traJ* mRNA expression in *E*. *coli* harboring the conjugative plasmids pTA-Mob 2.1 and pSC5.1 by qRT-PCR. The mean ± SE are given for six biological replicates normalized to the reference genes *rrsA* and *cysG*. Student’s t-test was used to carry out the pairwise comparison between pTA-Mob 2.1 and pSC5.1 and asterisks denote the significant difference between pairwise comparison (*, *P* < 0.05; **, *P* < 0.01).

**
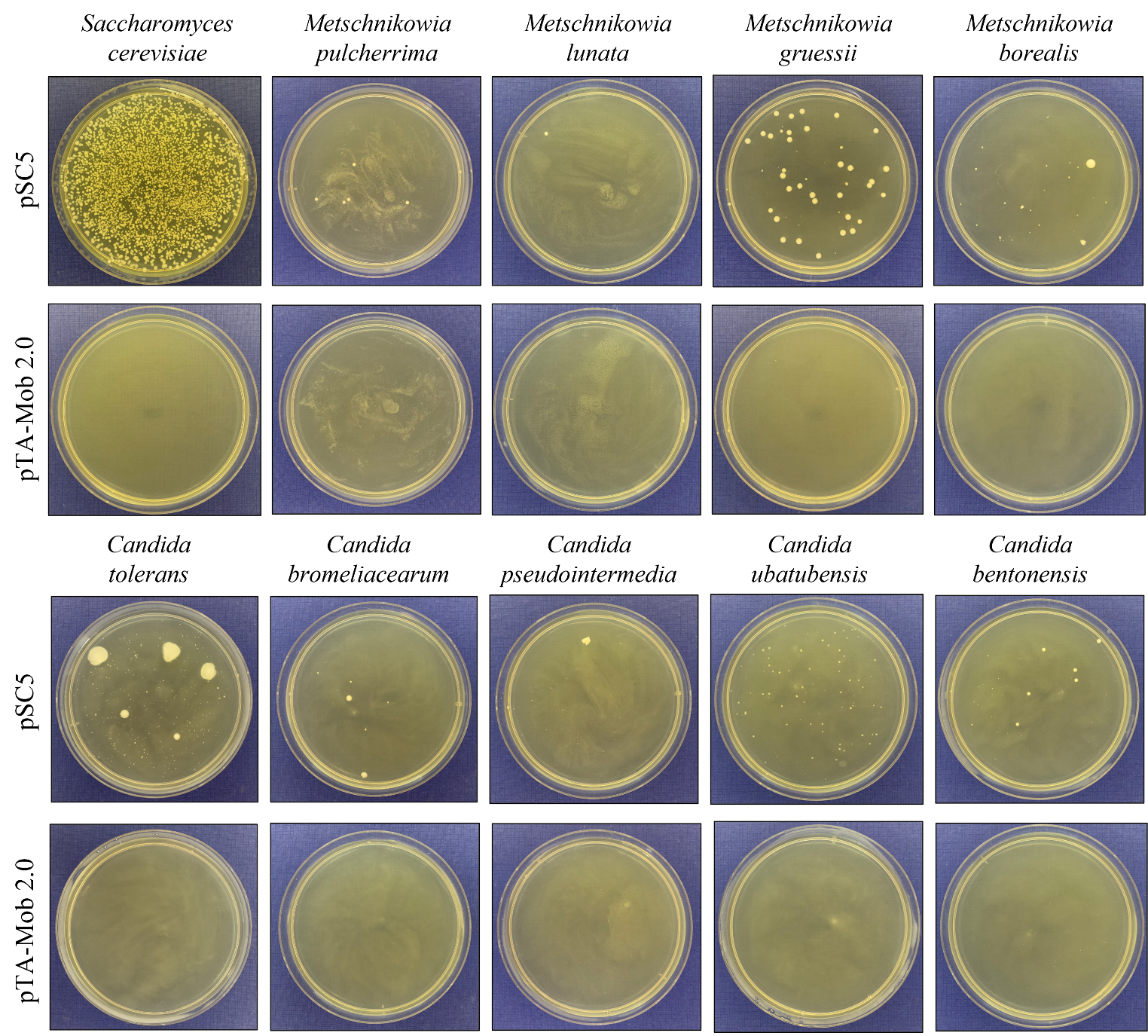
**

**Supplemental Figure S4. Bacterial conjugation from *E. coli* to diverse yeast species.** Representative plates of yeast transconjugants following conjugation from *E. coli* harboring either pSC5 or pTA-Mob 2.0, plated on YPAD media supplemented with nourseothricin (100 µg mL^-1^) and ampicillin (100 µg mL^-1^).


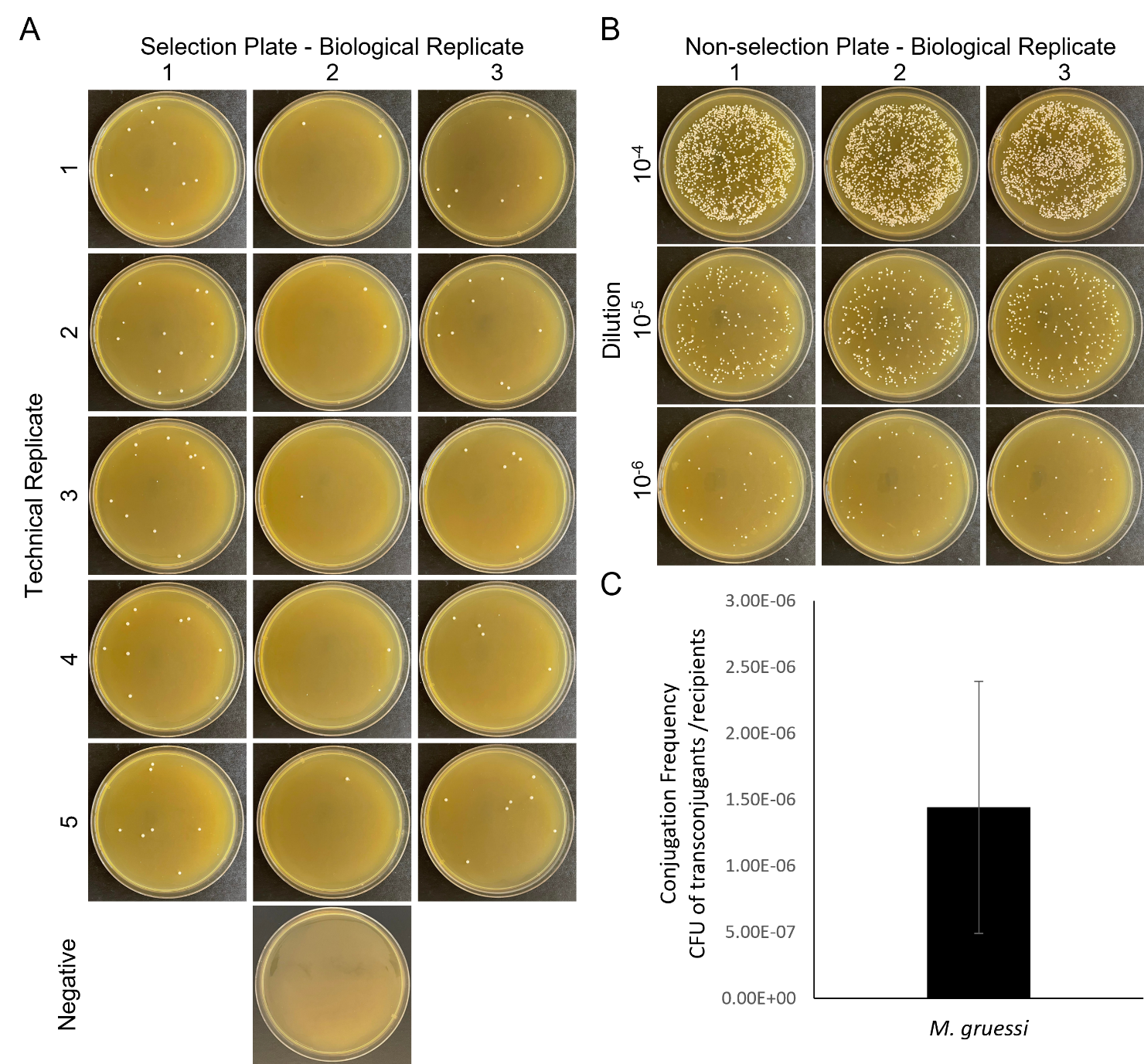


**Supplemental Figure S5.** **Conjugation frequency of pSC5 to *Metschnikowia gruessi*.** A) Three replicates of *M. gruessi* transconjugants plated on five YPDA plates supplemented with nourseothricin (100 µg mL^-1^) and ampicillin (100 µg mL^-1^). B) Dilution series (10^-3^ – 10^-5^) of *M. gruessi* plated on YPDA plates supplemented with ampicillin (100 µg mL^-1^). C) Average conjugation frequency of *M. gruessi* represents the mean ± standard deviation for three biological replicates.

**
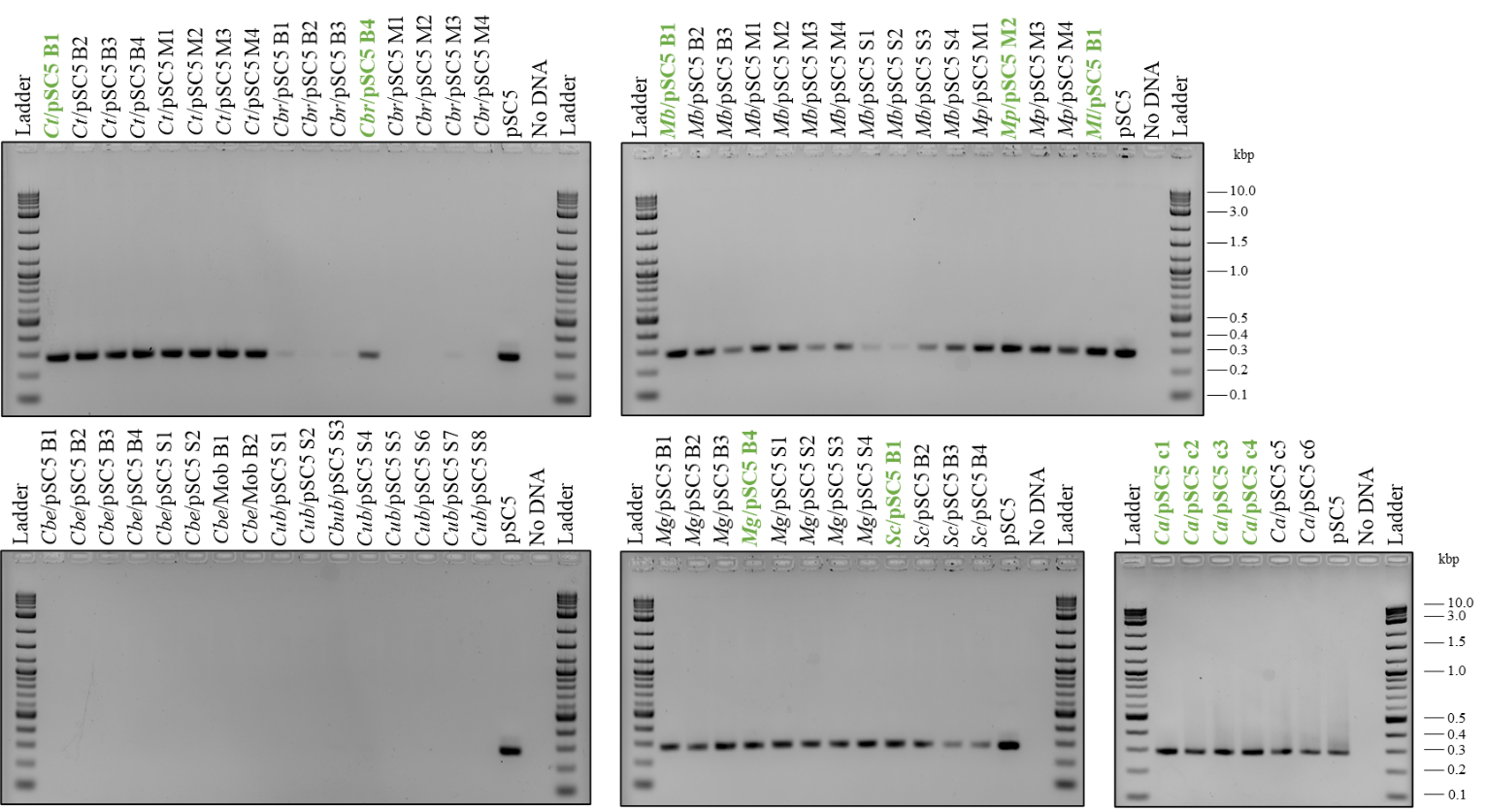
**

**Supplemental Figure S6. Genotyping transconjugants of diverse yeast species.** Genotyping of diverse yeast strains following conjugation with *E. coli* harboring pSC5. Multiplex PCR was performed to amplify the nourseothricin resistance gene of amplicon size 283-bp. *Ct* – *Candida tolerans*, *Cbr* – *Candida bromeliacearum*, *Mb* – *Metschnikowia borealis*, *Mp* – *Metschnikowia pulcherrima*, *Ml* – *Metschnikowia lunata*, *Cbe* – *Candida* aff. *bentonensis*, *Cub* – *Candida ubatubensis*, *Mg* – *Metschnikowia gruessi*, *Sc* – *Saccharomyces cerevisiae, Ca* – *Candida auris*, B – big colony, M – medium colony, and S – small colony*.* Clones highlighted in green were further analyzed with restriction enzyme digest and a phenotypic conjugation screen (**Figure 5**, **Supplemental Figure S7**). Ladder: NEB 2-log ladder.


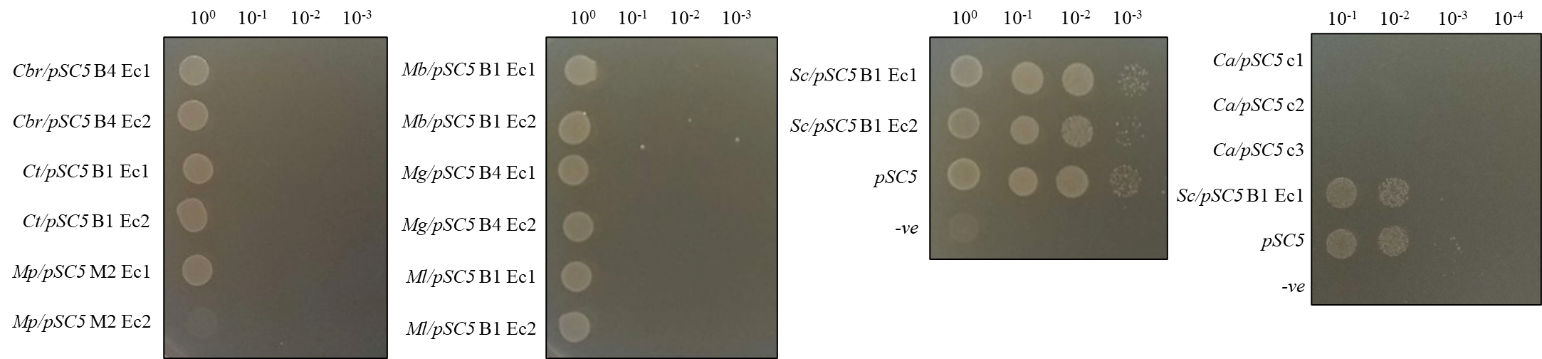


**Supplemental Figure S7. Phenotypic *E. coli* to *E. coli* conjugation screen of recovered transconjugant plasmids from diverse yeast species.** Selected recovered pSC5 plasmids from big diverse yeast colonies and *S. cerevisiae* colonies were transformed into *E. coli* and tested for conjugation to *E. coli* harboring pAGE1.0. Transconjugant *E. coli* were spot plated on LB plates supplemented with chloramphenicol (30 µg mL^-1^) and gentamicin (60 µg mL^-1^). *Ct* - *Candida tolerans*, *Cbr* - *Candida bromeliacearum*, *Cbe* - *Candida bentonensis*, *Cub* - *Candida ubatubensis*, *Mb* - *Metschnikowia borealis*, *Mp* - *Metschnikowia pulcherrima*, *Ml* - *Metschnikowia lunata*, *Mg* - *Metschnikowia gruessi*, *Sc* - *S. cerevisiae, Ca - Candida auris*, *Ec* - *Escherichia coli*, B – Big colony, M – Medium colony, -ve – Negative (*S. cerevisiae* only)*.*

**Supplementary Tables**

**Supplemental Table S1. Description of pTA-Mob 2.0 deletion plasmid library.** The fragment split by PCR amplification is listed, and regions of pTA-Mob 2.0 deleted are reported with respect to the 3’ end of backbone insertion re-indexed to position 1. Diagnostic multiplex primers used to screen deletion plasmids and their respective amplicon sizes are shown. Genes either completely or partially removed from pTA-Mob 2.0 in each deletion plasmid are listed, their location within pTA-Mob 2.0 is annotated in parentheses, and the primers used to remove them are provided.

| Deletion Plasmid | Fragment Split | Region Deleted | MPX Primers | Amplicon (bp) | Gene(s) Deleted  Or Partially Deleted | Additional primers for splitting the fragment of interest  Top primer pairs with the original reverse primer for the fragment of interest. Bottom primer pairs with the original forward primer of the fragment of interest. |
| --- | --- | --- | --- | --- | --- | --- |
| 1 | 2 | 574-1241 | F - aggcggtaaaggtgagcag  R - gaagcctgcgaagagttgc | 301 | *upf16.5* (574-1241) | gcggcagagatgaacacgaccatcagcggctgcacagcgccattgacccaggcgtgttcc  tgcgaggcagcggcctggtggaacacgcctgggtcaatggcgctgtgcagccgctgatg |
| 2 | 2 | 1216-2364 | F - ctctgtttatcggcagttcg  R - gtattcgtgcagggcaagat | 327 | *trfA1* (1216-2364) & *trfA2* (1216-2073) | tggctgctgaacccccagccggaactgaccccacaaggcctcaccctccttgcgggattg  tgccccggcgtgagtcggggcaatcccgcaaggagggtgaggccttgtggggtcagttcc |
| 3 | 2 | 1216-2073 | F - ctctgtttatcggcagttcg  R - tcgatggtccagcaagctac | 306 | *trfA2* (1216-2073) & *trfA1* (1216-2364) | tggctgctgaacccccagccggaactgaccccacaaggccagttcctcgcgtgtcgatgg  aggtttggcgaagtcgatgaccatcgacacgcgaggaactggccttgtggggtcagttcc |
| 4 | 2 | 2938-3249 | F - ctcggtgtcacgggtaagat  R - cacgacgtaggggttctgat | 393 | *trbA* (2884-3249) | atttttcaccaacatccttcgtctgctcgatgagcggggccgccaagggttagggcttgc  tagcggctaaagaaggaagtgcaagccctaacccttggcggccccgctcatcgagcagac |
| 5 | 2 | 3583-4479 | F - tgcgctttgacagttgtttt  R - ttcgtcagccagctctcata | 374 | *trbB* (3520-4479) | gaccatcaaggagcgggccaagcgcaagctggaacgcgacggagtatttccaatgacaac  tcagacggaacggaacagccgttgtcattggaaatactccgtcgcgttccagcttgcgct |
| 6 | 2 | 4492-4929 | F - attcaccgaaacccattgag  R - catgattcggaacgcataga | 349 | *trbC* (4492-4929) | cggccagtacatcaccaaaaccctgtaaggagtatttccatcatggctctgcgcacgatc  ttgcctgcgcgacggatggggatcgtgcgcagagccatgatggaaatactccttacaggg |
| 7 | 2 | 4932-5239 | F - ttcttccgaaccctgatctt  R - tccttggaacgatgcttttt | 304 | *trbD* (4932-5243) | atgccgtgcgtgcggtagcggctggacggctcgcctaatcatgatccaagcaattgcgat  cgccgaggcccgcgattgcaatcgcaattgcttggatcatgattaggcgagccgtccagc |
| 8 | 2/3 | 5244-7794 | F - gtcggtctgatcctgtggtt  R - gtctgcttttgcgacacaac | 338 | *trbE* (5240-7798) | ccgcgagaacaccaatagccaagggaagcaataccgatgaatgagttttgcagacacgat  tcttgaagatcaagcccttgatcgtgtctgcaaaactcattcatcggtattgcttccctt |
| 9 | 3 | 7799-8553 | F - cgtctctacgacctggcact  R - gtacccgcctcccacttct | 352 | *trbF* (7795-8553) | ccggggcctcgcccttgatgaatacctggaggcagcatgaggcactgaattatgaaaaag  aggaccaaagcaaacagttcctttttcataattcagtgcctcatgctgcctccaggtatt |
| 10 | 3 | 8565-9458 | F - ggtcagcaccgaaatcactt  R - agctgccgtacttggaggt | 319 | *trbG* (8565-9458) | gggacttctcctggtcgagacttctgtgaggcactgaattaccatgcgtaagattctgac  tggccgcgagtgcgatgacggtcagaatcttacgcatggtaattcagtgcctcacagaag |
| 11 | 3 | 9462-9944 | F - acggcaagaaaaccatcatc  R - tgaggtacatcggcatgttg | 331 | *trbH* (9462-9944) | cagccaggaccgcgtgaccatttcaagggggaactaaaccgccaatgagcgaagatcaaa  tggcgatgcgtccggtgccatttgatcttcgctcattggcggtttagttcccccttgaaa |
| 12 | 3 | 9949-11340 | F - agcaacctgtatcgcctgac  R - tcctcgatttgcttctggac | 313 | *trbI* (9949-11340) | atggtcccggccggcgcatgggttcggaaggagtaagccactccaaggagtaacttatga  aacattcttagcgagcttcttcataagttactccttggagtggcttactccttccgaacc |
| 13 | 3/4 | 11357-12133 | F - gcagatgatcgccaaaaact  R - gggcacttcttcaacagctc | 312 | *trbJ* (11357-12133) | ccctaccaggcgtttgactattaactccaaggagtaacttggggaggcgcgatgaagaaa  gcaactgcgatgaagttggatttcttcatcgcgcctccccaagttactccttggagttaa |
| 14 | 4 | 12145-12354 | F - caaatccgtggccttctg  R - acggtcaaggtccagaacag | 349 | *trbK* (12145-12354) | accgcgcaagcccgtctaagacctggtgaggggaggcgcgtgacgtatgaaaatccagac  ccgcgagcgcggcagctctagtctggattttcatacgtcacgcgcctcccctcaccaggt |
| 15 | 4 | 12361-13947 | F - gatctgccgaaggtcacg  R - cttgtggatgccgaagtacc | 329 | *trbL* (12361-13947) | aggcttcaagcccagcgaaaagaaagagtggtgatgacgtaacgactcttaggagctacg  gcttttttcagttgcatggtcgtagctcctaagagtcgttacgtcatcaccactctttct |
| 16 | 4 | 13971-14570 | F - gttcgacgacaacagccttt  R - aagaatgttggccggaatct | 324 | *trbM* (13971-14570) | agcccgcccaatcctgaaacgactcttaggagctacgaccggggaggggatagcgatgcc  gtgccagcagcttggcaaacggcatcgctatcccctccccggtcgtagctcctaagagtc |
| 17 | 4 | 14586-15290 | F - gccatgcctataccgacttc  R - tgcccagtaccagaagatca | 336 | *trbN* (14586-15290) | cgcctaccgtcggttcgagcggtaaggggaggggatagcgggaggaacggccgtttagcg  gaatgcccataggctttagccgctaaacggccgttcctcccgctatcccctccccttacc |
| 18 | 4 | 15321-15584 | F - ggcagctcatcatcaacaac  R - ccaggacgaaaacgaaaaga | 300 | *trbO* (15321-15584) | cacgccataaggaggaacggccgtttagcggctaaagcctcgccctgcagggcgttctta  tcatgccctcccccttggagtaagaacgccctgcagggcgaggctttagccgctaaacgg |
| 19 | 4 | 15621-16355 | F - aagcgccgtttgatcttct R - catggggatgttcagcagt | 321 | *trbP* (15621-16355) | atagcgccctgcagggcgttcttactccaagggggagggccctttgagattccaatatgc  ggtgcatttcttgagcaattgcatattggaatctcaaagggccctcccccttggagtaag |
| 20 | 4 | 16372-16791 | F - acgtactacccgctgcatct  R - gatagaacgcttcggtgtcc | 343 | *upf31.7* (16372-16791) | gcgggctacttgtttttcacctgacctttgagattccaatggggagggcggcggatgctg  aggaagcccttcaaccgtgtcagcatccgccgccctccccattggaatctcaaaggtcag |
| 21 | 4/5 | 16806-17501 | F - ataaccagctcgccatcaag  R - cagcaccaggaacatcgtc | 318 | *fiwA* (16806-17501) | cgcttgacgatgcgaagttctactgaggggagggcggcggctacaaccgtgcgcaaggcg  tacatacatcctccctaatgcgccttgcgcacggttgtagccgccgccctcccctcagta |
| 22 | 5 | 17537-18199 | F - cgagctgctgaacaaggttt  R - gagccaggtcaaacgagtgt | 303 | *upf32.8* (17537-18199) | tctgactacaaccgtgcgcaaggcgcattagggaggatgtgaaaagccgggcactgcccg  cgcagcagcaaaaataaagccgggcagtgcccggcttttcacatcctccctaatgcgcct |
| 23 | 5 | 18223-18882 | F - gactacaccgagggggaaag  R - gagaaggacaccgaccgtta | 331 | *parA1* (18223-18882) & *parA2* (18223-18846) | gggacgcgaaaaggtgagaaaagccgggcactgcccggctttccacggctcgacggcgtg  tcggcctctggtccgatccgcacgccgtcgagccgtggaaagccgggcagtgcccggctt |
| 24 | 5 | 18223-18846 | F - gactacaccgagggggaaag  R - gtgggtcaacatggagctg | 326 | *parA2* (18223-18846) & *parA1* (18223-18882) | gggacgcgaaaaggtgagaaaagccgggcactgcccggctccttcgtccctccggttgtt  ggaaatggcgacgcgagagcaacaaccggagggacgaaggagccgggcagtgcccggctt |
| 25 | 5 | 18843-19688 | F - tctcctggcgttcaagattc  R - tccgtcatgtcgattgtcag | 217 | *parB* (18843-19376) & *parC* (19373-19666) & *parA1* (18223-18882) & *parA2* (18223-18846) | tgctcgtccgtactggcgcgcaggtagatgcgggcgacctggcgcattacagcaatacgc  gcgctaggcgcatttaaattgcgtattgctgtaatgcgccaggtcgcccgcatctacctg |
| 26 | 5 | 19373-19666 | F - tgcttgtctaccagcacgtc  R - tccgtcatgtcgattgtcag | 318 | *parC* (19373-19666) | agcgcggcagcggcgcgcagcatggcgtagcttcggcgctttgggcctagtctagccggc  gtattgctgtaatgcgccatgccggctagactaggcccaaagcgccgaagctacgccatg |
| 27 | 5 | 19817-20068 | F - catggcgcattacagcaata  R - gggaaagagttcgctcatgt | 304 | *parD* (19817-20068) & *parE* (20065-20376) | tgtgctaatgtggttacgtgtattttatggaggttatccacggcctacatcctcacggct  cgtagatcggcttcggcctcagccgtgaggatgtaggccgtggataacctccataaaata |
| 28 | 6 | 23490-24286 | F - cgaggttggtggtaatcgtt  R - gccgcatagtgtagccagat | 328 | *istB* (23489-24286) | ggaacatcgaccactgagtgcctatgaggagctgttgtgacgaaagtatcttagcgggca  accgttatttgccattttcatgcccgctaagatactttcgtcacaacagctcctcatagg |
| 29 | 6 | 24763-24993 | F - agactagccaccaccattgc  R - acggaggtagcagcagaaaa | 328 | *aphA* (24763-24993) | gaacgatattgatcgagaagagccctgcgcagccgctgccggcgatgccccctcgacctc  tcctgaacgcctccctgatcgaggtcgagggggcatcgccggcagcggctgcgcagggct |
| 30 | 6 | 25077-25367 | F - tgcaatttcatctcctgctg  R - cgcaacgtctaccagttcag | 308 | *traA* (25077-25367) | ctcacaaagaaagccgggcaatgcccggctttttctgctgacgcctcctagatcgagcgc  aaggccgagcagaaacgctcgcgctcgatctaggaggcgtcagcagaaaaagccgggcat |
| 31 | 6 | 25377-25815 | F - gttgacgatgatgctgttgg  R - gccaggtgagaagtgctgtc | 347 | *traB* (25375-25815) | gaaaatcgtgcgggtacgcctcgatgttcatacgcctcctacgctctagttctccttagt  gcaacgccgcgcgagaacctactaaggagaactagagcgtaggaggcgtatgaacatcga |
| 32 | 6/7 | 25831-29016 | F - accttcatggtcagccattc  R - gagttcgacccggaagaag | 314 | *traC1* (25831-29016) & *traC2* (25831-28071) | gtgccccgatctgtactttgttcatacgctctagttctccagccaattacctcccgtcat  cacgacgaccgcggccgccaatgacgggaggtaattggctggagaactagagcgtatgaa |
| 33 | 6 | 25831-28071 | F - accttcatggtcagccattc  R - atcaaggcgctacaagagga | 308 | *traC2* (25831-28071) & *traC1* (25831-29016) | gtgccccgatctgtactttgttcatacgctctagttctccagttgccccctgcgcaggct  aacgacccagcaggccatcgagcctgcgcagggggcaactggagaactagagcgtatgaa |
| 34 | 7 | 29023-29286 | F - tgtaacgcttcccggtagtc  R - agtctccgagctgcacaagt | 340 | *traD* (29023-29286) | cctgctcgtggaacggctttttgacctctgccatagccaacgctttcactcctggttggt  acagcaaaggccgtaacggcaccaaccaggagtgaaagcgttggctatggcagaggtcaa |
| 35 | 7 | 29292-31505 | F - accaggtcaatgtcgctctc  R - gccgaggttctgcttatgtc | 342 | *traE* (29292-31505) | aagtcgtcctgctgcacggtcttgggatcattcatcgcttatatccccctaccctcacca  tcatcaggccggttctgacctggtgagggtagggggatataagcgatgaatgatcccaag |
| 36 | 7 | 31520-32049 | F - tagtcctccgggtctagcaa  R - gcgacatggttgtgtacgtc | 345 | *traF* (31520-32053) | gcgattacaaggcgttcaaattgcatatatccccctaccctcatatcgtgatcccctccc  gacggccaccgtcgaggaaggggaggggatcacgatatgagggtagggggatatatgcaa |
| 37 | 7 | 32054-33953 | F - gcttttggtggtgttgacct  R - tgattatgttggtgcgctgt | 316 | *traG* (32050-33957) | cgatggcgacgtacttggtgaggcgctggaagcggctcattcatctactcctacctcggg  gcgaggctcccttaaaactacccgaggtaggagtagatgaatgagccgcttccagcgcct |
| 38 | 7/8 | 33958-36131 | F - tttttcgcccgtatctgtg  R - gtatccaacggcgtcagaat | 505 | *traH* (34253-34612) & *traI* (33954-36152) | cccgtatctgtggccccacggcgttgtttcggttcttcatggggacgtgcttggcaatca  aacggccggggggtgcgcgtgattgccaagcacgtccccatgaagaaccgaaacaacgc |
| 39 | 7 | 34253-34612 | F - gcaccagaatctcgtcgttt  R - gtcaacggcacagcagagt | 316 | *traH* (34253-34612) & *traI* (33954-36152) | tcggcaacatatttctcggccgccgcgatctgttcgggctgtgcttgctccttcgtcagt  gagcgccgccgtcaagaactactgacgaaggagcaagcacagcccgaacagatcgcggcg |
| 40 | 8 | 36149-36190 | F - ctcggtcttgccttgctc  R - gtatccaacggcgtcagaat | 488 | *traX* (36149-36190) *traI* (33954-36152) & *traJ* (36187-36558) | tcgctcttcttgatggagcgcatggggacgtgcttggcaaggctctgccctcgggcggac  gcaaggtcatgatgggcgtggtccgcccgagggcagagccttgccaagcacgtccccatg |
| 41 | 8 | 36187-36558 | F - gcgaagtcgctcttcttgat  R - gctctttggcatcgtctctc | 333 | *traJ* (36187-36558) & *traX* (36149-36190) | aatcacgcgcaccccccggccgttttagcggctaaaaaagccgcttgccgaattctgacg  ttccttggtgtatccaacggcgtcagaattcggcaagcggcttttttagccgctaaaacg |
| 42 | 8 | 36796-37199 | F - tcgctataatgaccccgaag  R - gttgcgcgagttaatttcgt | 338 | *traK* (36796-37200) | atgcaggaaattactgaactgaggggacaggcgagagacgatggcgaaaattcacatggt  ccccgcccttgccctgcaaaaccatgtgaattttcgccatcgtctctcgcctgtcccctc |
| 43 | 8 | 37201-37921 | F - aacggggaaggtcaagttct  R - tgatggtatgcaggatcagc | 335 | *traL* (37200-37925) | caccttcaacccaacaccggacaaaaaggatctactgtaaatgagcgaccagattgaaga  ccgcaatctcccggatcagctcttcaatctggtcgctcatttacagtagatcctttttgt |
| 44 | 8 | 37926-38359 | F - ggcaagagctttgagcagat  R - tgtcaaacaagcccagctaa | 312 | *traM* (37922-38359) | cggcctgtttgaacagctcgacgcggcggccgtgctatgaatcgcagaggcgcagatgaa  gcccggcaacgccgggctttttcatctgcgcctctgcgattcatagcacggccgccgcgt |
| 45 | 8 | 38411-39758 | F - ggcgaaggtaatgaaggaca  R - tgcgaaaagcatcacctatg | 348 | *upf54.4* (38412-39758) | gcagatgaaaaagcccggcgttgccgggcttgtttttgcggacgcctccctttttagccg  cgcactcgttagagttttagcggctaaaaagggaggcgtccgcaaaaacaagcccggcaa |
| 46 | 8/9 | 40292-41218 | F - tggttctcggtctggacaat  R - acctgccgttaagtcgagaa | 305 | *kfrA* (40292-41218) | ttgttccatctattttagtgaactgcgttcgatttatcaggttggctccggtaattggta  ggtaagagtattattattcttaccaattaccggagccaacctgataaatcgaacgcagtt |
| 47 | 9 | 41932-42444 | F - ggatagcttgcaacatcagga  R - acccggacaagctgaaaaag | 295 | *korF* (41941-42444) | cttgcaacatcaggagccgtttcttttgttcgtcagtcattcttcggtcctccttgtagc  gccgcacagacaacggttccgctacaaggaggaccgaagaatgactgacgaacaaaagaa |
| 48 | 9 | 44843-45792 | F - ttggttgaacatagcggtga  R - gttcagacgacgctcatcaa | 313 | *klaC* (44843-45796) | aaaacaaaagcccggaaaccgggctttcgtctcttgccgctcatacggactcctgttggg  ccagcccgtgcgcgagctggcccaacaggagtccgtatgagcggcaagagacgaaagccc |
| 49 | 9/10 | 45793-46875 | F - ctggtcgctgaatgtcgat  R - gctcatcaccaccaagaaca | 392 | *klaB* (45793-46929) & *klaC* (44843-45796) | aggccgcctacctgggcgaaaacatcggtgtttgtggcatgtcgaaggcgacgatagggg  aacgacgcacgacgccaaggcccctatcgtcgccttcgacatgccacaaacaccgatgtt |
| 50 | 10 | 46947-47720 | F - catgtcgaaggcgacgatag  R - ttcgatggatgtttgctttg | 317 | *klaA* (46947-47720) | tgcgtcgttttcagtgcgttcatagggttctcccgccgtgtcgatacaccctcgcggtgg  tccatcgaaaagcaattaacccaccgcgagggtgtatcgacacggcgggagaaccctatg |
| 51 | 10 | 47843-48157 | F - gacagctcgttgagggaatc  R - ggtactgaccgcactcacct | 332 | *kleF1* (47843-48157) & *kleF2* (47843-48013) | ccgctaaaatttggggacaggtcatttacagaaagccagctgctcaaagctccttgaagg  ggagggtcaagcaagcggccccttcaaggagctttgagcagctggctttctgtaaatgac |
| 52 | 10 | 47839-48013 | F - caaaacctcccccttcaatc  R - tttgagcaatgccaagacag | 318 | *kleF2* (47843-48013) & *kleF1* (47843-48157) | ttagccgctaaaatttggggacaggtcatttacagaaagcgccccagccctcggcctccg  atcggcgtactggttcaatccggaggccgagggctggggcgctttctgtaaatgacctgt |
| 53 | 10 | 48185-48508 | F - ctccggattgaaccagtacg  R - aagtcaccaagtgggtcgag | 341 | *kleE* (48185-48508) | cctgtcttggcattgctcaaagctccttgaaggggccgcttgcttgaccctccacggcga  ctggacgaattgaacacgcatcgccgtggagggtcaagcaagcggccccttcaaggagct |
| 54 | 10 | 48620-48838 | F - cacgcctgggaacttgataa  R - gaatgctattgccgagaagc | 319 | *kleD* (48620-48838) | atcgaggtcaagcgccccggagaaatccggggcgtcatcccatcatcccctggcgtcagt  ggcgaaattccgggccggtcactgacgccaggggatgatgggatgacgccccggatttct |
| 55 | 10 | 48854-49084 | F - ctcgacccacttggtgactt  R - caggggaaaggtgttttcaa | 324 | *kleC* (48854-49084) | agttcaccctatctcctacttgcatcatcatcccctggcgcggattggcctccggtaatt  gccgggtagattcccaggtcaattaccggaggccaatccgcgccaggggatgatgatgca |
| 56 | 10 | 49237-49452 | F - gcttctcggcaatagcattc  R - actggtccacccaggaagtc | 338 | *kleB* (49237-49452) | gaggcgtcatgcttgaaaacacctttcccctggcgtgcaaggccctatctccttgagaga  ggcccggctacggtcgggcctctctcaaggagatagggccttgcacgccaggggaaaggt |
| 57 | 10 | 49501-49734 | F - ggctaagggtcgaaatgga  R - actttacgccaagggagagg | 336 | *kleA* (49501-49734) | ctccttgagagaggcccgaccgtagccgggcctcgttccgcggggttgatcctccggttg  gggcacttcgcccaggtcagcaaccggaggatcaaccccgcggaacgaggcccggctacg |

**Supplemental Table S2. List of primers used to amplify the assembly fragments and genotype the plasmids created in this study.**

| Fragment | Primer | Primer Sequence (5’ to 3’) | Expected size (bp) | Template |
| --- | --- | --- | --- | --- |
| **M1** | | | | |
| F1 | F | tgccgccgcgcgcatggtcgtaatgggaccgatagcccgt | 5782 | pTA-Mob 2.0 |
|  | R | tttaacctacttcctttggttccgggggatctcgcgactc |  |  |
| F2 | F | atcgaagagaagcaggacga | 6373 | pTA-Mob 2.0 |
|  | R | tgctggtccatgaagatgaa |  |  |
| F3 | F | tcgagctgatgtttgacgac | 6137 | pTA-Mob 2.0 |
|  | R | ggacttgaggttgctctgct |  |  |
| F4 | F | gtggacattggtttcagcaa | 3693 | pTA-Mob 2.0 |
|  | R | gcgagaacctactaaggagaactagagcgtacgtgcttcgaaccactcggagggacggtt |  |  |
| F5 | F | aaccgtccctccgagtggttcgaagcacgtacgctctagttctccttagtaggttctcgc | 3476 | pTA-Mob 2.0 |
|  | R | aagcgatgaatgatcccaag |  |  |
| F6 | F | gagcaatggatagccgatgt | 6206 | pTA-Mob 2.0 |
|  | R | aagcgatgaatgatcccaag |  |  |
| F7 | F | tgtaacgcttcccggtagtc | 6295 | pTA-Mob 2.0 |
|  | R | cattgcaaagcgactgatgt |  |  |
| F8 | F | gatccgctccttgaactctg | 6259 | pTA-Mob 2.0 |
|  | R | aggcccttgccaatgaat |  |  |
| F9 | F | ttctttgaatgcgcgggcgtcctggtgagcgtagtccagc | 6000 | pTA-Mob 2.0 |
|  | R | cgttcccgcctgcccctgattggcccgctgatcgaccgct |  |  |
| F10 | F | aatgttgcaaggcgatcag | 5745 | pTA-Mob 2.0 |
|  | R | agccctcccgtatcgtagtt |  |  |
| **M2** | | | | |
| F1 | F | tgccgccgcgcgcatggtcgtaatgggaccgatagcccgt | 5782 | pTA-Mob 2.0 |
|  | R | tttaacctacttcctttggttccgggggatctcgcgactc |  |  |
| F2 | F | atcgaagagaagcaggacga | 6373 | pTA-Mob 2.0 |
|  | R | tgctggtccatgaagatgaa |  |  |
| F3 | F | tcgagctgatgtttgacgac | 6137 | pTA-Mob 2.0 |
|  | R | ggacttgaggttgctctgct |  |  |
| F4 | F | ggaccaggcgcagtccaccatcaacggcctgatgagcgcc | 6000 | pTA-Mob 2.0 |
|  | R | atcggcgtgaagcccaacagggcca |  |  |
| F5 | F | gtggacattggtttcagcaa | 6273 | pTA-Mob 2.0 |
|  | R | agctcatgcatcacaacagc |  |  |
| F6 | F | gagcaatggatagccgatgt | 6206 | pTA-Mob 2.0 |
|  | R | aagcgatgaatgatcccaag |  |  |
| F7 | F | tgtaacgcttcccggtagtc | 6295 | pTA-Mob 2.0 |
|  | R | cattgcaaagcgactgatgt |  |  |
| F8 | F | gatccgctccttgaactctg | 6259 | pTA-Mob 2.0 |
|  | R | aggcccttgccaatgaat |  |  |
| F9 | F | ttctttgaatgcgcgggcgtcctgg | 4224 | pTA-Mob 2.0 |
|  | R | tgaacacgcatcgccgtggagggtcaagcagcggcaagagacgaaagcccggtttccggg |  |  |
| F10 | F | cccggaaaccgggctttcgtctcttgccgctgcttgaccctccacggcgatgcgtgttca | 2664 | pTA-Mob 2.0 |
|  | R | agccctcccgtatcgtagtt |  |  |
| **M3** | | | | |
| F1 | F | tgccgccgcgcgcatggtcgtaatgggaccgatagcccgt | 5782 | pTA-Mob 2.0 |
|  | R | tttaacctacttcctttggttccgggggatctcgcgactc |  |  |
| F2 | F | atcgaagagaagcaggacga | 6373 | pTA-Mob 2.0 |
|  | R | tgctggtccatgaagatgaa |  |  |
| F3 | F | tcgagctgatgtttgacgac | 6137 | pTA-Mob 2.0 |
|  | R | ggacttgaggttgctctgct |  |  |
| F4 | F | ggaccaggcgcagtccaccatcaacggcctgatgagcgcc | 6000 | pTA-Mob 2.0 |
|  | R | atcggcgtgaagcccaacagggcca |  |  |
| F5 | F | gtggacattggtttcagcaa | 3693 | pTA-Mob 2.0 |
|  | R | gcgagaacctactaaggagaactagagcgtacgtgcttcgaaccactcggagggacggtt |  |  |
| F6 | F | aaccgtccctccgagtggttcgaagcacgtacgctctagttctccttagtaggttctcgc | 3476 | pTA-Mob 2.0 |
|  | R | aagcgatgaatgatcccaag |  |  |
| F7 | F | tgtaacgcttcccggtagtc | 6295 | pTA-Mob 2.0 |
|  | R | cattgcaaagcgactgatgt |  |  |
| F8 | F | gatccgctccttgaactctg | 6259 | pTA-Mob 2.0 |
|  | R | aggcccttgccaatgaat |  |  |
| F9 | F | ttctttgaatgcgcgggcgtcctggtgagcgtagtccagc | 6000 | pTA-Mob 2.0 |
|  | R | cgttcccgcctgcccctgattggcccgctgatcgaccgct |  |  |
| F10 | F | aatgttgcaaggcgatcag | 5745 | pTA-Mob 2.0 |
|  | R | agccctcccgtatcgtagtt |  |  |
| **M4** | | | | |
| F1 | F | tgccgccgcgcgcatggtcgtaatgggaccgatagcccgt | 5782 | pTA-Mob 2.0 |
|  | R | tttaacctacttcctttggttccgggggatctcgcgactc |  |  |
| F2 | F | atcgaagagaagcaggacga | 6373 | pTA-Mob 2.0 |
|  | R | tgctggtccatgaagatgaa |  |  |
| F3 | F | tcgagctgatgtttgacgac | 6137 | pTA-Mob 2.0 |
|  | R | ggacttgaggttgctctgct |  |  |
| F4 | F | ggaccaggcgcagtccaccatcaacggcctgatgagcgcc | 6000 | pTA-Mob 2.0 |
|  | R | atcggcgtgaagcccaacagggcca |  |  |
| F5 | F | gtggacattggtttcagcaa | 3476 | pTA-Mob 2.0 |
|  | R | ggttctgacctggtgagggtagggggatatacgtgcttcgaaccactcggagggacggtt |  |  |
| F6 | F | aaccgtccctccgagtggttcgaagcacgtatatccccctaccctcaccaggtcagaacc | 3655 | pTA-Mob 2.0 |
|  | R | tgtaacgcttcccggtagtc |  |  |
| F7 | F | gatccgctccttgaactctg | 6259 | pTA-Mob 2.0 |
|  | R | aggcccttgccaatgaat |  |  |
| F8 | F | ttctttgaatgcgcgggcgtcctggtgagcgtagtccagc | 6000 | pTA-Mob 2.0 |
|  | R | cgttcccgcctgcccctgattggcccgctgatcgaccgct |  |  |
| F9 | F | aatgttgcaaggcgatcag | 5745 | pTA-Mob 2.0 |
|  | R | agccctcccgtatcgtagtt |  |  |
| **M5** | | | | |
| F1 | F | tgccgccgcgcgcatggtcgtaatgggaccgatagcccgt | 11593 | M3C1 |
|  | R | tgctggtccatgaagatgaa |  |  |
| F2 | F | tcgagctgatgtttgacgac | 8884 | M3C1 |
|  | R | tcatcaggccggttctgacctggtgagggtagggggatatcgctatcccctccccttacc |  |  |
| F3 | F | ggtaaggggaggggatagcgatatccccctaccctcaccaggtcagaaccggcctgatga | 3675 | M3C1 |
|  | R | cattgcaaagcgactgatgt |  |  |
| F4 | F | gatccgctccttgaactctg | 6253 | M3C1 |
|  | R | aggcccttgccaatgaat |  |  |
| F5 | F | ttctttgaatgcgcgggcgtcctggtgagcgtagtccagc | 10447 | M3C1 |
|  | R | agccctcccgtatcgtagtt |  |  |
| **M6** | | | | |
| F1 | F | aaaacaaaagcccggaaaccgggctttcgtctcttgccgccggggttgatcctccggttg | 12900 | M3C1 |
|  | R | tgctggtccatgaagatgaa |  |  |
| F2 | F | tcgagctgatgtttgacgac | 11800 | M3C1 |
|  | R | tcatcaggccggttctgacctggtgagggtagggggatatcgctatcccctccccttacc |  |  |
| F3 | F | ggtaaggggaggggatagcgatatccccctaccctcaccaggtcagaaccggcctgatga | 3675 | M3C1 |
|  | R | cattgcaaagcgactgatgt |  |  |
| F4 | F | gatccgctccttgaactctg | 6253 | M3C1 |
|  | R | aggcccttgccaatgaat |  |  |
| F5 | F | ttctttgaatgcgcgggcgtcctggtgagcgtagtccagc | 4262 | M3C1 |
|  | R | gggcacttcgcccaggtcagcaaccggaggatcaaccccggcggcaagagacgaaagccc |  |  |
| **M7** | | | | |
| F1 | F | tgccgccgcgcgcatggtcgtaatgggaccgatagcccgt | 5132 | M3C1 |
|  | R | tgccccggcgtgagtcggggcaatcccgcaaggagggtgaccgcttgccctcatctgtta |  |  |
| F2 | F | ctggccggctaccgccggcgtaacagatgagggcaagcggtcaccctccttgcgggattg | 3819 | M3C1 |
|  | R | tgctggtccatgaagatgaa |  |  |
| F3 | F | tcgagctgatgtttgacgac | 8884 | M3C1 |
|  | R | tcatcaggccggttctgacctggtgagggtagggggatatcgctatcccctccccttacc |  |  |
| F4 | F | ggtaaggggaggggatagcgatatccccctaccctcaccaggtcagaaccggcctgatga | 3675 | M3C1 |
|  | R | cattgcaaagcgactgatgt |  |  |
| F5 | F | gatccgctccttgaactctg | 6253 | M3C1 |
|  | R | aggcccttgccaatgaat |  |  |
| F6 | F | ttctttgaatgcgcgggcgtcctggtgagcgtagtccagc | 10447 | M3C1 |
|  | R | agccctcccgtatcgtagtt |  |  |
| **M8** | | | | |
| F1 | F | aaaacaaaagcccggaaaccgggctttcgtctcttgccgccggggttgatcctccggttg | 6610 | M3C1 |
|  | R | tgccccggcgtgagtcggggcaatcccgcaaggagggtgaccgcttgccctcatctgtta |  |  |
| F2 | F | ctggccggctaccgccggcgtaacagatgagggcaagcggtcaccctccttgcgggattg | 11686 | M3C1 |
|  | R | tgacctggtgagggtagggggatatcgctatcccctccccggtcgtagctcctaagagtc |  |  |
| F3 | F | agcccgcccaatcctgaaacgactcttaggagctacgaccggggaggggatagcgatatc | 3710 | M3C1 |
|  | R | cattgcaaagcgactgatgt |  |  |
| F4 | F | gatccgctccttgaactctg | 6253 | M3C1 |
|  | R | aggcccttgccaatgaat |  |  |
| F5 | F | ttctttgaatgcgcgggcgtcctggtgagcgtagtccagc | 4262 | M3C1 |
|  | R | gggcacttcgcccaggtcagcaaccggaggatcaaccccggcggcaagagacgaaagccc |  |  |
| **M3C1_F1** | | | | |
| F1 | F | tgccgccgcgcgcatggtcgtaatgggaccgatagcccgt | 11593 | M3C1 |
|  | R | tgctggtccatgaagatgaa |  |  |
| F2 | F | tcgagctgatgtttgacgac | 11800 | M3C2 |
|  | R | atcggcgtgaagcccaacagggcca |  |  |
| F3 | F | gtggacattggtttcagcaa | 6500 | M3C2 |
|  | R | aagcgatgaatgatcccaag |  |  |
| F4 | F | tgtaacgcttcccggtagtc | 11797 | M3C2 |
|  | R | aggcccttgccaatgaat |  |  |
| F5 | F | ttctttgaatgcgcgggcgtcctggtgagcgtagtccagc | 10447 | M3C2 |
|  | R | agccctcccgtatcgtagtt |  |  |
| **M3C1_F2** | | | | |
| F1 | F | tgccgccgcgcgcatggtcgtaatgggaccgatagcccgt | 11593 | M3C2 |
|  | R | tgctggtccatgaagatgaa |  |  |
| F2 | F | tcgagctgatgtttgacgac | 11800 | M3C1 |
|  | R | atcggcgtgaagcccaacagggcca |  |  |
| F3 | F | gtggacattggtttcagcaa | 6500 | M3C2 |
|  | R | aagcgatgaatgatcccaag |  |  |
| F4 | F | tgtaacgcttcccggtagtc | 11797 | M3C2 |
|  | R | aggcccttgccaatgaat |  |  |
| F5 | F | ttctttgaatgcgcgggcgtcctggtgagcgtagtccagc | 10447 | M3C2 |
|  | R | agccctcccgtatcgtagtt |  |  |
| **M3C1_F3** | | | | |
| F1 | F | tgccgccgcgcgcatggtcgtaatgggaccgatagcccgt | 11593 | M3C2 |
|  | R | tgctggtccatgaagatgaa |  |  |
| F2 | F | tcgagctgatgtttgacgac | 11800 | M3C2 |
|  | R | atcggcgtgaagcccaacagggcca |  |  |
| F3 | F | gtggacattggtttcagcaa | 6500 | M3C1 |
|  | R | aagcgatgaatgatcccaag |  |  |
| F4 | F | tgtaacgcttcccggtagtc | 11797 | M3C2 |
|  | R | aggcccttgccaatgaat |  |  |
| F5 | F | ttctttgaatgcgcgggcgtcctggtgagcgtagtccagc | 10447 | M3C2 |
|  | R | agccctcccgtatcgtagtt |  |  |
| **M3C1_F4** | | | | |
| F1 | F | tgccgccgcgcgcatggtcgtaatgggaccgatagcccgt | 11593 | M3C2 |
|  | R | tgctggtccatgaagatgaa |  |  |
| F2 | F | tcgagctgatgtttgacgac | 11800 | M3C2 |
|  | R | atcggcgtgaagcccaacagggcca |  |  |
| F3 | F | gtggacattggtttcagcaa | 6500 | M3C2 |
|  | R | aagcgatgaatgatcccaag |  |  |
| F4 | F | tgtaacgcttcccggtagtc | 11797 | M3C1 |
|  | R | aggcccttgccaatgaat |  |  |
| F5 | F | ttctttgaatgcgcgggcgtcctggtgagcgtagtccagc | 10447 | M3C2 |
|  | R | agccctcccgtatcgtagtt |  |  |
| **M3C1_F5** | | | | |
| F1 | F | tgccgccgcgcgcatggtcgtaatgggaccgatagcccgt | 11593 | M3C2 |
|  | R | tgctggtccatgaagatgaa |  |  |
| F2 | F | tcgagctgatgtttgacgac | 11800 | M3C2 |
|  | R | atcggcgtgaagcccaacagggcca |  |  |
| F3 | F | gtggacattggtttcagcaa | 6500 | M3C2 |
|  | R | aagcgatgaatgatcccaag |  |  |
| F4 | F | tgtaacgcttcccggtagtc | 11797 | M3C2 |
|  | R | aggcccttgccaatgaat |  |  |
| F5 | F | ttctttgaatgcgcgggcgtcctggtgagcgtagtccagc | 10447 | M3C1 |
|  | R | agccctcccgtatcgtagtt |  |  |
| **pTA-Mob 2.0 Tp** | | | | |
| F1 | F | tgccgccgcgcgcatggtcgtaatgggaccgatagcccgt | 5752 | pTA-Mob 2.0 |
|  | R | tttaacctacttcctttggttccgggggatctcgcgactc |  |  |
| F2 | F | atcgaagagaagcaggacga | 6374 | pTA-Mob 2.0 |
|  | R | tgctggtccatgaagatgaa |  |  |
| F3 | F | tcgagctgatgtttgacgac | 6137 | pTA-Mob2.0 |
|  | R | ggacttgaggttgctctgct |  |  |
| F4 | F | ggaccaggcgcagtccaccatcaacggcctgatgagcgcc/ | 6000 | pTA-Mob 2.0 |
|  | R | atcggcgtgaagcccaacagggcca |  |  |
| F5 | F | gtggacattggtttcagcaa | 6273 | pTA-Mob 2.0 |
|  | R | agctcatgcatcacaacagc |  |  |
| F6 | F | gagcaatggatagccgatgt | 6206 | pTA-Mob 2.0 |
|  | R | aagcgatgaatgatcccaag |  |  |
| F7 | F | tgtaacgcttcccggtagtc | 6295 | pTA-Mob 2.0 |
|  | R | cattgcaaagcgactgatgt |  |  |
| F8a | F | gatccgctccttgaactctg | 1881 | pTA-Mob 2.0 |
|  | R | atccaacggcgtcagccgagggcaagcggatggctgatgaaaccaagccaaccaggaagg |  |  |
| F8b | F | ccttcctggttggcttggtttcatcagccatccgcttgccctcggctgacgccgttggat | 4435 | pTA-Mob 2.0 |
|  | R | aggcccttgccaatgaat |  |  |
| F9 | F | ttctttgaatgcgcgggcgtcctggtgagcgtagtccagc | 6000 | pTA-Mob 2.0 |
|  | R | cgttcccgcctgcccctgattggcccgctgatcgaccgct |  |  |
| F10 | F | aatgttgcaaggcgatcag | 5745 | pTA-Mob 2.0 |
|  | R | agccctcccgtatcgtagtt |  |  |
| **pTA-Mob 2.0 To** | | | | |
| F1 | F | tgccgccgcgcgcatggtcgtaatgggaccgatagcccgt | 5752 | pTA-Mob 2.0 |
|  | R | tttaacctacttcctttggttccgggggatctcgcgactc |  |  |
| F2 | F | atcgaagagaagcaggacga | 6374 | pTA-Mob 2.0 |
|  | R | tgctggtccatgaagatgaa |  |  |
| F3 | F | tcgagctgatgtttgacgac | 6137 | pTA-Mob2.0 |
|  | R | ggacttgaggttgctctgct |  |  |
| F4 | F | ggaccaggcgcagtccaccatcaacggcctgatgagcgcc | 6000 | pTA-Mob 2.0 |
|  | R | atcggcgtgaagcccaacagggcca |  |  |
| F5 | F | gtggacattggtttcagcaa | 6273 | pTA-Mob 2.0 |
|  | R | agctcatgcatcacaacagc |  |  |
| F6 | F | gagcaatggatagccgatgt | 6206 | pTA-Mob 2.0 |
|  | R | aagcgatgaatgatcccaag |  |  |
| F7 | F | tgtaacgcttcccggtagtc | 6295 | pTA-Mob 2.0 |
|  | R | cattgcaaagcgactgatgt |  |  |
| F8a | F | gatccgctccttgaactctg | 1821 | pTA-Mob 2.0 |
|  | R | gcagcccacctatcaaggtgtactgccttccagacgaacgaagagcgattgaggaaaagg |  |  |
| F8b | F | taggccgacaggctcatgccggccgccgccgccttttcctcaatcgctcttcgttcgtct | 4526 | pTA-Mob 2.0 |
|  | R | aggcccttgccaatgaat |  |  |
| F9 | F | ttctttgaatgcgcgggcgtcctggtgagcgtagtccagc | 6000 | pTA-Mob 2.0 |
|  | R | cgttcccgcctgcccctgattggcccgctgatcgaccgct |  |  |
| F10 | F | aatgttgcaaggcgatcag | 5745 | pTA-Mob 2.0 |
|  | R | agccctcccgtatcgtagtt |  |  |
| **Sequencing primers to check mutations in *traJ* region** | | | | |
| F1 | F | gtttcagcaggccgcccagg |  |  |
|  | R | cgctgcataaccctgcttcg |  |  |
| **pSC5** | | | | |
| F1 | F | tgccgccgcgcgcatggtcgtaatgggaccgatagcccgt | 7280 | pTA-Mob 2.0-NAT |
|  | R | tttaacctacttcctttggttccgggggatctcgcgactc |  |  |
| F2 | F | atcgaagagaagcaggacga | 6373 | M3C1 |
|  | R | tgctggtccatgaagatgaa |  |  |
| F3 | F | tcgagctgatgtttgacgac | 11800 | M3C1 |
|  | R | atcggcgtgaagcccaacagggcca |  |  |
| F4 | F | gtggacattggtttcagcaa | 3692 | M3C1 |
|  | R | caagcattgggttccgtatctaaccatgaccgtgcttcgaaccactcggagggacggttt |  |  |
| F5 | F | aaaccgtccctccgagtggttcgaagcacggtcatggttagatacggaacccaatgcttg | 2573 | pGMO1 |
|  | R | aggagaactagagcgtaattaccctgttatccctacaaacaccctttcaatgggcttcga |  |  |
| F6 | F | cattgaaagggtgtttgtagggataacagggtaattacgctctagttctccttagtaggt | 3477 | M3C1 |
|  | R | aagcgatgaatgatcccaag |  |  |
| F7 | F | tgtaacgcttcccggtagtc | 11797 | M3C1 |
|  | R | aggcccttgccaatgaat |  |  |
| F8 | F | ttctttgaatgcgcgggcgtcctggtgagcgtagtccagc | 10447 | M3C1 |
|  | R | cgttcccgcctgcccctgattggcccgctgatcgaccgct |  |  |
| F9 | F | aatgttgcaaggcgatcag | 5745 | MV3C1 |
|  | R | agccctcccgtatcgtagtt |  |  |
| **pSC5GGv1** | | | | |
| F1 | F | tgccgccgcgcgcatggtcgtaatgggaccgatagcccgt | 6112 | pSC5 |
|  | R | taattagcatttttcgctttattctgttgtcgagatcttctccttgcaggttcaacaact |  |  |
| F2 | F | aagatctcgacaacagaataaagcgaaaaatgctaataatgcactaacactcaggcctc  (To domesticate BsaI site) | 7196 | pSC5 |
|  | R | tgctggtccatgaagatgaa |  |  |
| F3 | F | tcgagctgatgtttgacgac | 6137 | pSC5 |
|  | R | ggacttgaggttgctctgct |  |  |
| F4 | F | ggaccaggcgcagtccaccatcaacggcctgatgagcgcc | 6000 | pSC5 |
|  | R | atcggcgtgaagcccaacagggcca |  |  |
| F5 | F | gtggacattggtttcagcaa | 11926 | pSC5 |
|  | R | caatgtctgatgcaatatggacaattggtttcttggtctcattaccctgttatccctaca |  |  |
| F6 | F | accttcgggtgggcctttctgcgtttataggtctcatgcttacgctctagttctccttag | 1187 | pSC5 |
|  | R | cgaattgaaacggagggcgacaagaagggcgagaagtcgggcttctacgtcggccacctc |  |  |
| F7 | F | cgacttctcgcccttcttgtcgccctccgtttcaattcggtgcttcttgccgtccatga  (To domesticate BsaI site) | 8239 | pSC5 |
|  | R | cattgcaaagcgactgatgt |  |  |
| F8 | F | gatccgctccttgaactctg | 6256 | pSC5 |
|  | R | aggcccttgccaatgaat |  |  |
| F9 | F | aatgttgcaaggcgatcag | 4928 | pSC5 |
|  | R | agccctcccgtatcgtagtt |  |  |
| F10 | F | attgaaagggtgtttgtagggataacagggtaatgagaccaagaaaccaattgtccatat  (To amplify RFP Gene) | 1276 | pAGE2.0-i |
|  | R | Gagaacctactaaggagaactagagcgtaagcatgagacctataaacgcagaaaggccca  (To amplify RFP Gene) |  |  |
| ***ShBle* fragment for Golden Gate assembly** | | | | |
| ***ShBle*** gene | F | ggtctcagtaatatcaagcttg | 1063 | pRS32 (From Shapiro lab) |
|  | R | taggtctcaagcaactggatggcg |  |  |
| **HindII fragment for Golden Gate assembly** | | | | |
| HindII toxic gene | F | ggtctcagtaaggcgcgcccttggcagaacatatccatcgcgtccgccatctccagcagc | 1618 | pUC57 HindII plasmid (Synthesized vector) |
|  | R | ggtctcagatttatcttcgtttcctgcaggtttttgttctgtgcagttgggttaagaata |  |  |
| ***A.* *laidlawii* toxic gene fragments for Golden Gate assembly** | | | | |
| 1^st^ half of Al toxic gene | F | ggtctcagtaaggtaatgcttggcatgttcatatagatggtttaacagatcattataaag | 2072 | *A. laidlawii* strain PG-8A gDNA |
|  | R | ggtctcatagtgcattaaatcctggaggcgttaatttacttctatgtgcttcaaaggctt |  |  |
| *ACT1* intron | F | ggtctcaactagtatgttctagcgcttgcaccatcccatttaactgtaagaagaattgca | 327 | *S*. *cerevisiae* gDNA |
|  | R | ggtctcactaaacatataatatagcaacaaaaagaatgaagcaatcgatgttagtacatg |  |  |
| 2^nd^ half of Al toxic gene | F | ggtctcattagtctaatgaaggtttacttcaatattacgcttcattaaatggtttaactg | 1061 | *A. laidlawii* strain PG-8A gDNA |
|  | R | ggtctcaagcaggaccataagaagtccgaaaaactattaatctgtccaaaatgtttaata |  |  |
| **Genotyping *yNAT* marker** | | | | |
| *NAT* gene | F | tccagttgatccaccattga | 283 |  |
|  | R | caaccacaaatgaccagcac |  |  |
| **pSC5GGv2** | | | | |
| F1 | F | tgccgccgcgcgcatggtcgtaatgggaccgatagcccgt | 4235 | pSC5GGv1 C8 |
|  | R | atatggacaattggtttcttggtctcattacatatatacacatgtatatatatcgtatgc |  |  |
| F2 | F | tgggcctttctgcgtttataggtctcatgctgtatacctatgaatgtcagtaagtatgta | 3149 | pSC5GGv1 C8 |
|  | R | tttaacctacttcctttggttccgggggatctcgcgactc |  |  |
| F3 | F | taggagtgcggttggaacgt | 6177 | pSC5GGv1 C8 |
|  | R | tgctggtccatgaagatgaa |  |  |
| F4 | F | tcgagctgatgtttgacgac | 6137 | pSC5GGv1 C8 |
|  | R | ggacttgaggttgctctgct |  |  |
| F5 | F | ggaccaggcgcagtccaccatcaacggcctgatgagcgcc | 6000 | pSC5GGv1 C8 |
|  | R | atcggcgtgaagcccaacagggcca |  |  |
| F6 | F | gtggacattggtttcagcaa | 6300 | pSC5GGv1 C8 |
|  | R | aggagaactagagcgtaattaccctgttatccctacaaacaccctttcaatgggcttcga |  |  |
| F7 | F | cattgaaagggtgtttgtagggataacagggtaattacgctctagttctccttagtaggt | 3512 | pSC5GGv1 C8 |
|  | R | aagcgatgaatgatcccaag |  |  |
| F8 | F | tgtaacgcttcccggtagtc | 6295 | pSC5GGv1 C8 |
|  | R | cattgcaaagcgactgatgt |  |  |
| F9 | F | gatccgctccttgaactctg | 6253 | pSC5GGv1 C8 |
|  | R | aggcccttgccaatgaat |  |  |
| F10 | F | ttctttgaatgcgcgggcgtcctggtgagcgtagtccagc | 6000 | pSC5GGv1 C8 |
|  | R | cgttcccgcctgcccctgattggcccgctgatcgaccgct |  |  |
| F11 | F | aatgttgcaaggcgatcag | 4928 | pSC5GGv1 C8 |
|  | R | agccctcccgtatcgtagtt |  |  |
| F12 | F | gcatacgatatatatacatgtgtatatatgtaatgagaccaagaaaccaattgtccatat  (To amplify mRFP Gene) | 1276 | pAGE2.0-i |
|  | R | tacatacttactgacattcataggtatacagcatgagacctataaacgcagaaaggccca  (To amplify mRFP Gene) |  |  |
| **pTA-Mob 2.1** | | | | |
| F1 | F | tgccgccgcgcgcatggtcgtaatgggaccgatagcccgt | 5259 | pTA-Mob 2.0 |
|  | R | gggagtatctggctgggccaacgttccaaccgcactcctaccggccagcctcgcagagca |  |  |
| F2 | F | gcggtgctcaacgggaatcctgctctgcgaggctggccggtaggagtgcggttggaacgt | 6177 | pTA-Mob 2.0 |
|  | R | tgctggtccatgaagatgaa |  |  |
| F3 | F | tcgagctgatgtttgacgac | 6137 | pTA-Mob 2.0 |
|  | R | ggacttgaggttgctctgct |  |  |
| F4 | F | ggaccaggcgcagtccaccatcaacggcctgatgagcgcc | 6000 | pTA-Mob 2.0 |
|  | R | atcggcgtgaagcccaacagggcca |  |  |
| F5 | F | gtggacattggtttcagcaa | 6289 | pTA-Mob 2.0 |
|  | R | agctcatgcatcacaacagc |  |  |
| F6 | F | gagcaatggatagccgatgt | 6204 | pTA-Mob 2.0 |
|  | R | aagcgatgaatgatcccaag |  |  |
| F7 | F | tgtaacgcttcccggtagtc | 6295 | pTA-Mob 2.0 |
|  | R | cattgcaaagcgactgatgt |  |  |
| F8 | F | gatccgctccttgaactctg | 6256 | pTA-Mob 2.0 |
|  | R | aggcccttgccaatgaat |  |  |
| F9 | F | ttctttgaatgcgcgggcgtcctggtgagcgtagtccagc | 6000 | pTA-Mob 2.0 |
|  | R | cgttcccgcctgcccctgattggcccgctgatcgaccgct |  |  |
| F10 | F | aatgttgcaaggcgatcag | 4928 | pTA-Mob 2.0 |
|  | R | agccctcccgtatcgtagtt |  |  |
| **pSC5.1** | | | | |
| F1 | F | tgccgccgcgcgcatggtcgtaatgggaccgatagcccgt | 6757 | pSC5 |
|  | R | cacgcgacaagcacgagcgagatatcccaatcaagctagtccggccagcctcgcagagca |  |  |
| F2 | F | gcggtgctcaacgggaatcctgctctgcgaggctggccggtaggagtgcggttggaacgt | 6177 | pSC5 |
|  | R | tgctggtccatgaagatgaa |  |  |
| F3 | F | tcgagctgatgtttgacgac | 6137 | pSC5 |
|  | R | ggacttgaggttgctctgct |  |  |
| F4 | F | ggaccaggcgcagtccaccatcaacggcctgatgagcgcc | 6000 | pSC5 |
|  | R | atcggcgtgaagcccaacagggcca |  |  |
| F5 | F | gtggacattggtttcagcaa | 9812 | pSC5 |
|  | R | aagcgatgaatgatcccaag |  |  |
| F6 | F | tgtaacgcttcccggtagtc | 6295 | pSC5 |
|  | R | cattgcaaagcgactgatgt |  |  |
| F7 | F | gatccgctccttgaactctg | 6256 | pSC5 |
|  | R | aggcccttgccaatgaat |  |  |
| F8 | F | ttctttgaatgcgcgggcgtcctggtgagcgtagtccagc | 10549 | pSC5 |
|  | R | agccctcccgtatcgtagtt |  |  |
| **qPCR primers** | | | | |
| *rrsA* gene | F | ctcttgccatcggatgtgccca | 106 | pTA-Mob 2.1 and pSC5.1 cDNA |
|  | R | ccagtgtggctggtcatcctctca |  |  |
| *cysG* gene | F | ttgtcggcggtggtgatgtc | 136 | pTA-Mob 2.1 and pSC5.1 cDNA |
|  | R | atgcggtgaactgtggaataaacg |  |  |
| *traJ* gene | F | acgacgcccgtgattttgtag | 109 | pTA-Mob 2.1 and pSC5.1 cDNA |
|  | R | gccttccagacgaacgaaga |  |  |

**Supplemental Table S3. Conjugation phenotype of pTA-Mob 2.0 deletion plasmid library.** Conjugation phenotypes of pTA-Mob 2.0 deletion strains displayed as the ratio of the number of deletion plasmids transconjugants relative to pTA-Mob 2.0 transconjugants. All clones were tested using 1 biological and 3 technical replicates, except for deletion plasmid 32 **C2*** where 6 biological and 1 – 3 technical replicates were used. Additional replicas were performed for plasmid 32 **C2*** due to the high variations in the initial experiments. Deletions were categorized as either non-essential (green; 0.51 – 5.98), semi-essential (light-green; 0.06 – 0.50), or essential (red & orange; 0 – 0.05) based on their bacterial conjugation ratio. Contradictory results for clones for the same genes could be due to mutations introduced during the PCR fragment amplification or yeast assembly. N/D: Not done.

| **Deletion Plasmid** | **Clone name** | **Ratio for Clone 1** | **Ratio for Clone 2** |
| --- | --- | --- | --- |
| **1** | **C1, C2** | **0.36** | **1.04** |
| **2** | **C1, C5** | **1.25** | **0.00** |
| **3** | **C2, C3** | **1.90** | **0.00** |
| **4** | **C2, C3** | **0.00** | **0.00** |
| **5** | **C1, C2** | **0.00** | **0.00** |
| **6** | **C2, C3** | **0.00** | **0.00** |
| **7** | **C2, C3** | **0.00** | **0.00** |
| **8** | **C4, C5** | **0.00** | **0.00** |
| **9** | **C2, C8** | **0.00** | **0.00** |
| **10** | **C2, C2.1** | **0.00** | **0.00** |
| **11** | **C2, C3** | **0.01** | **0.01** |
| **12** | **C2, C5** | **0.00** | **0.00** |
| **13** | **C4, C5** | **0.00** | **0.01** |
| **14** | **C1, C2** | **2.37** | **3.60** |
| **15** | **C1, C5** | **0.00** | **0.00** |
| **16** | **C1, C5** | **3.09** | **0.01** |
| **17** | **C2, C3** | **N/D** | **0.12** |
| **18** | **C2, C3** | **1.31** | **1.11** |
| **19** | **C4, C5** | **N/D** | **0.15** |
| **20** | **C2, C3** | **0.54** | **0.39** |
| **21** | **C4, C5** | **0.31** | **0.98** |
| **22** | **C2, C3** | **0.53** | **0.64** |
| **23** | **C1, C2** | **0.51** | **0.46** |
| **24** | **C3, C16** | **0.63** | **1.03** |
| **25** | **C1, C2.1** | **N/D** | **N/D** |
| **26** | **C2.2, C3** | **0.52** | **1.39** |
| **27** | **C1, C2** | **2.06** | **0.11** |
| **28** | **C2** | **N/D** | **0.34** |
| **29** | **C2, C5** | **0.98** | **0.00** |
| **30** | **C6, C7** | **4.36** | **1.22** |
| **31** | **C1, C2** | **0.57** | **1.47** |
| **32** | **C1, C2*** | **0.75** | **5.98*** |
| **33** | **C3, C6** | **0.20** | **0.24** |
| **34** | **C1, C3** | **0.00** | **0.51** |
| **35** | **C1.1, C6** | **0.33** | **1.09** |
| **36** | **C1, C2** | **0.00** | **0.00** |
| **37** | **C2** | **0.00** | **N/D** |
| **38** | **C2, C3** | **0.00** | **0.00** |
| **39** | **C2, C8** | **0.00** | **0.24** |
| **40** | **C1, C5** | **0.00** | **0.00** |
| **41** | **C5, C7** | **0.00** | **0.00** |
| **42** | **C2, C4** | **N/D** | **0.00** |
| **43** | **C2, C4** | **0.00** | **0.00** |
| **44** | **C3, C5** | **0.00** | **0.00** |
| **45** | **C9, C10** | **0.00** | **0.04** |
| **46** | **C1.1, C10** | **0.00** | **0.22** |
| **47** | **C1, C5** | **0.65** | **1.49** |
| **48** | **C1** | **0.05** | **N/D** |
| **49** | **C1, C5** | **0.32** | **0.41** |
| **50** | **C4, C5** | **0.39** | **0.67** |
| **51** | **C3, C5** | **N/D** | **N/D** |
| **52** | **C1, C2** | **1.81** | **0.61** |
| **53** | **C2** | **N/D** | **0.69** |
| **54** | **C1, C2** | **0.00** | **0.00** |
| **55** | **C1, C5** | **0.43** | **0.41** |
| **56** | **C2, C3** | **0.46** | **0.60** |
| **57** | **C1, C5** | **0.65** | **1.03** |

**Supplemental Table S4. Whole plasmid sequencing of minimal conjugative plasmid 3 (M3C1 and M3C2).** Mutations in M3C1 and M3C2 identified by next-generation sequencing and alignment to the reference sequence: pTA-Mob 2.0. Nucleotide numbering begins at the forward primers of Fragment 1. Note: The 4,912 bp deletion in M3C1 and M3C2 was deleted intentionally.

| Plasmid | Fragment | Plasmid position | Nucleotide Mutation | Amino Acid Mutation | Gene |
| --- | --- | --- | --- | --- | --- |
| M3C1 | Fragment 1 | 3,342 | 1 bp insertion (T) |  |  |
| M3C1 | Fragment 2 | 14,111 | G_731_ → T | R_244_ → L | *trbF* |
| M3C1 | Fragment 2 | 20,676 | G_505_ → T | D_169_ → Y | *trbN* |
| M3C1 | Fragment 3 | 25,788 | C_138_ → A |  |  |
| M3C1 | Fragment 3 | 26,489 | 4,912 bp Deletion |  | *URA3* |
| M3C1 | Fragment 4 | 37,156 | T_77_ → G | E_26_ → A | *traJ* |
| M3C1 | Fragment 4 | 37,242 – 37,246 | GAATT → CTCGG |  |  |
| M3C2 | Fragment 3 | 25,787 | C_138_ → A |  | *parE* |
| M3C2 | Fragment 3 | 26,488 | 4,912 bp Deletion |  | *URA3* |
| M3C2 | Fragment 4 | 37,458 | G → T |  |  |
| M3C2 | Fragment 5 | 48,347 | A_47_ → G | L_16_ → S | *klaA* |

**Supplemental Table S5. *Cis*- and *trans*- conjugation of superior conjugative plasmid (pSC5).** *S. cerevisiae* transconjugant concentrations following bacterial conjugation of pSC5 compared to pTA-Mob 2.0 in either *cis-* (self-transmissible) or *trans-* (mobilization of a secondary plasmid – pAGE2.0.T) and *E. coli* transconjugant colony counts following bacterial conjugation of pSC5 compared to pTA-Mob 2.0 in *cis-* from *E. coli* (**Figure 4.**). Results are shown as colony-forming units per mL (CFU mL^-1^) for four biological replicates each with two technical replicates.

| Configuration | Plasmid | Rep 1  (CFU mL^-1^) | Rep 2  (CFU mL^-1^) | Rep 3  (CFU mL^-1^) | Rep 4  (CFU mL^-1^) | Average (CFU mL^-1^) |
| --- | --- | --- | --- | --- | --- | --- |
| *Cis*  (*S. cerevisiae*) | pTA-Mob 2.0 | 3.1 x 10^3^ | 4.2 x 10^3^ | 3.6 x 10^3^ | 3.8 x 10^3^ | 3.7 x 10^3^ |
|  | pSC5 | 1.9 x 10^6^ | 1.3 x 10^5^ | 1.3 x 10^5^ | 1.0 x 10^5^ | 1.4 x 10^5^ |
| *Trans*  (*S. cerevisiae*) | pTA-Mob 2.0 | 6.6 x 10^2^ | 5.7 x 10^2^ | 1.3 x 10^3^ | 2.8 x 10^3^ | 1.3 x 10^3^ |
|  | pSC5 | 5.3 x 10^4^ | 8.3 x 10^4^ | 8.5 x 10^4^ | 6.2 x 10^4^ | 7.1 x 10^4^ |
| *Cis*  (*E*. *coli*) | pTA-Mob 2.0 | 2.2 x 10^6^ | 1.0 x 10^6^ | 2.2 x 10^6^ | 2.7 x 10^6^ | 2.1 x 10^6^ |
|  | pSC5 | 1.0 x 10^6^ | 1.4 x 10^6^ | 1.0 x 10^6^ | 1.2 x 10^6^ | 1.2 x 10^6^ |

**Supplemental Table S6. Recipient yeast cell concentrations used in conjugation experiments of Figure 4.** Counts of *S. cerevisiae* colonies formed on the non-selective plate (1 × YPAD supplemented with ampicillin 100 µg mL^-1^) following the bacterial conjugation of pSC5 and pTA-Mob 2.0 in *cis-* and *trans*- configuration. Colony-forming units are presented per mL (CFU mL^-1^) for four biological replicates each with two technical replicates.

| Configuration | Plasmid | Rep 1  (CFU mL^-1^) | Rep 2  (CFU mL^-1^) | Rep 3  (CFU mL^-1^) | Rep 4  (CFU mL^-1^) | Average  (CFU mL^-1^) |
| --- | --- | --- | --- | --- | --- | --- |
| *Cis* | pTA-Mob 2.0 | 1.3 x 10^7^ | 1.5 x 10^7^ | 3.9 x 10^7^ | 4.7 x 10^7^ | 2.9 x 10^7^ |
|  | pSC5 | 6.7 x 10^7^ | 6.9 x 10^7^ | 1.1 x 10^8^ | 1.1 x 10^8^ | 9.1 x 10^7^ |
| *Trans* | pTA-Mob 2.0 | 8.0 x 10^6^ | 4.0 x 10^6^ | 4.3 x 10^7^ | 3.7 x 10^7^ | 2.3 x 10^7^ |
|  | pSC5 | 1.2 x 10^7^ | 1.5 x 10^7^ | 9.5 x 10^7^ | 9.3 x 10^7^ | 5.4 x 10^7^ |

**Supplemental Table S7. *S. cerevisiae* cell viability following conjugation with different *E. coli* strains.** *S. cerevisiae* cell concentrations obtained by hemocytometer (all cells) or plating on non-selective (1 × YPAD supplemented with ampicillin 100 µg mL^-1^) plates (live cells) following a 3-hour incubation at 30⁰C alone or with *E. coli* either harboring no plasmid, pTA-Mob 2.0, or pSC5.

| Donor (D) / Recipient (R) | Hemocytometer Cell Count (Cells mL^-1^) | Colony Count (CFU mL^-1^) | |
| --- | --- | --- | --- |
|  |  | Technical replicas | Average |
| D: no *E. coli*  R: *S. cerevisiae* | 5.08 x 10^8^ | 2.01 x 10^8^ | 2.08 x 10^8^ |
|  |  | 2.27 x 10^8^ |  |
|  |  | 1.97 x 10^8^ |  |
| D: *E. coli* Epi300  R: *S. cerevisiae* | 6.14 x 10^8^ | 2.05 x 10^8^ | 2.19 x 10^8^ |
|  |  | 2.37 x 10^8^ |  |
|  |  | 2.15 x 10^8^ |  |
| D: *E. coli* Epi300 with  pTA-Mob 2.0  R: *S. cerevisiae* | 2.56 x 10^8^ | 1.4 x 10^7^ | 1.63 x 10^7^ |
|  |  | 1.0 x 10^7^ |  |
|  |  | 2.5 x 10^7^ |  |
| D: *E. coli* Epi300 with  pSC5  R: *S. cerevisiae* | 4.06 x 10^8^ | 1.41 x 10^8^ | 1.44 x 10^8^ |
|  |  | 1.45 x 10^8^ |  |
|  |  | 1.47 x 10^8^ |  |

**Supplemental Table S8. *S. cerevisiae* cell viability following conjugation with different *S. meliloti* strains.** *S. cerevisiae* cell counts by hemocytometer (all cells) or plating on non-selective (1 × YPAD supplemented with ampicillin 100 µg mL^-1^) plates (live cells) following a 3-hour incubation at 30⁰C alone or with *S. meliloti* either harboring pTA-Mob 2.0 or pSC5.

| Donor (D) / Recipient (R) | Hemocytometer Cell Count (Cells mL^-1^) | | Colony Count (CFU mL^-1^) | |
| --- | --- | --- | --- | --- |
|  | Biological replicas | Average | Biological replicas | Average |
| D: no *S. meliloti*  R: *S. cerevisiae* | 3.04 x 10^8^ | 3.04 x 10^8^ | 1.27 x 10^8^ | 1.27 x 10^8^ |
| D: *S. meliloti* Rm4126 R^-^ with pTA-Mob 2.0  R: *S. cerevisiae* | 3.16 x 10^8^ | 3.07 x 10^8^ | 9.70 x 10^7^ | 8.47 x 10^7^ |
|  | 3.48 x 10^8^ |  | 1.10 x 10^8^ |  |
|  | 2.58 x 10^8^ |  | 4.70 x 10^7^ |  |
| D: *S. meliloti* Rm4126 R^-^ with pSC5  R: *S. cerevisiae* | 3.84 x 10^8^ | 4.11 x 10^8^ | 1.85 x 10^8^ | 1.60 x 10^8^ |
|  | 4.52 x 10^8^ |  | 1.67 x 10^8^ |  |
|  | 3.98 x 10^8^ |  | 1.27 x 10^8^ |  |

**Supplemental Table S9. *S. cerevisiae* transconjugant colony count following conjugation with *S. meliloti*.** Colony counts of *S. cerevisiae* transconjugant re-suspension (Total volume = 2 mL) following bacterial conjugation with three biological replicates of *S. meliloti* (Rm4126) harboring either pTA-Mob 2.0 or pSC5, plated on complete synthetic yeast media lacking histidine and supplemented with ampicillin (100 µg mL^-1^) (**Supplemental Figure S3**).

|  |  | Colony Count (CFU) | |
| --- | --- | --- | --- |
|  | Plasmid | 100 µL plated | 50 µL plated |
| Experiment 1 | pTA-Mob 2.0 #1 | 114 | 53 |
|  | pTA-Mob 2.0 #2 | 108 | 34 |
|  | pTA-Mob 2.0 #3 | 92 | 46 |
|  | pSC5 #1 | 2514 | 1341 |
|  | pSC5 #2 | 2399 | 1254 |
|  | pSC5 #3 | 2504 | 1220 |
| Experiment 2 | pTA-Mob 2.0 #1 | 147 | 68 |
|  | pTA-Mob 2.0 #2 | 123 | 54 |
|  | pTA-Mob 2.0 #3 | 87 | 39 |
|  | pSC5 #1 | 1044 | 546 |
|  | pSC5 #2 | 2145 | 1159 |
|  | pSC5 #3 | 572 | 260 |

**Supplemental Table S10. Yeast transconjugant colony counts for the conjugation-based antifungal experiment (Figure 7).** *S. cerevisiae* transconjugant colony counts following bacterial conjugation with *E. coli* harboring pAGE2.0.T and either pSC5, pSC5-toxic1, pSC5-toxic2, or pSC5-toxic3. Note: Colonies were counted from plating 100 µL of undiluted (1 X) and diluted (10 X) yeast transconjugant re-suspension (2 mL) on synthetic yeast media lacking either histidine or tryptophan. Colonies were counted manually. tmtc – too many to count; Rep – replicate.

| Plasmid | | Yeast Selection Marker (CFU) | | | | | | | |  |
| --- | --- | --- | --- | --- | --- | --- | --- | --- | --- | --- |
|  |  | *HIS3* | | | | *TRP1* | | | | Ratio *HIS3*/*TRP1* |
|  |  | Rep 1 | Rep 2 | Rep 3 | Average | Rep 1 | Rep 2 | Rep 3 | Average |  |
| 1 X | pSC5 | tmtc | tmtc | 592 | 592.00 | tmtc | tmtc | 302 | 302.0 | 1.960 |
|  | pSC5-toxic1 | tmtc | tmtc | 230 | 230.00 | tmtc | tmtc | 264 | 264.0 | 0.871 |
|  | pSC5-toxic2 | 25 | 17 | 5 | 15.67 | 594 | tmtc | 169 | 381.5 | 0.041 |
|  | pSC5-toxic3 | 2 | 1 | 0 | 1.00 | 428 | tmtc | 188 | 308.0 | 0.003 |
| 10 X | pSC5 | 208 | 436 | 55 | 233 | 174 | 262 | 33 | 156.3 | 1.490 |
|  | pSC5-toxic1 | 62 | 105 | 27 | 64.7 | 107 | 149 | 23 | 93 | 0.695 |
|  | pSC5-toxic2 | 4 | 1 | 0 | 1.7 | 66 | 72 | 18 | 52 | 0.032 |
|  | pSC5-toxic3 | 0 | 0 | 0 | 0 | 50 | 107 | 17 | 58 | 0 |

**Supplemental Methods**

**1.1. Bacterial Conjugation**

**1.1.1. *E. coli* to *E. coli* – *Cis-* Configuration**

*E*. *coli* donor and recipient strains were prepared as in **Methods and Materials 2.5** except the donor strains were resuspended in 5 mL of ice-cold 10% glycerol, and 500 µL aliquots were prepared in 1.5 mL Eppendorf tubes. To assess conjugation of pSC5 between bacteria, two donor strains of *E. coli* harboring either pSC5 or pTA-Mob 2.0 and an *E. coli* recipient strain harboring pAGE1.0 (chloramphenicol 15 µg mL^-1^; [Brumwell et al. 2019](https://paperpile.com/c/wwpjWu/bmyG); **Table 1**) were prepared and stored in the -80°C freezer. On the day of the conjugation, conjugation plates (20 mL, LB media with 1.5% agar) were prepared, and tubes containing the *E. coli* strains were removed from the freezer and thawed on ice. Once thawed, 10 µL of the donor *E. coli* strain was added to 100 µL of the recipient *E. coli* strain and mixed by pipetting prior to being transferred to the plate and spread evenly. Plates were incubated at 30°C for 90 minutes and then were scraped with 1.5 mL of sterile double distilled water and mixed thoroughly by vortexing for 5 seconds. A dilution series (10^-1^ – 10^-8^) was created in a 96-well plate and 100 μL of dilutions 10^-1^ – 10^-4^ were plated on selection plates (25 mL, LB media with 1.5% agar supplemented with chloramphenicol 15 µg mL^-1^ and gentamicin 40 µg mL^-1^). On non-selective plates (LB media with 1.5% agar supplemented with chloramphenicol 15 µg mL^-1^) 100 μL of dilutions 10^-1^ – 10^-8^ were plated. Plates were incubated at 37°C overnight and the following morning the colonies were counted, and conjugation frequency was calculated (transconjugant CFU / recipient CFU).

**1.1.2. *S. meliloti* to *S. cerevisiae* – *Cis-* Orientation**

*S. meliloti* was prepared similarly to *E. coli*; overnight cultures of a single colony were diluted to OD_600_ of 0.1 in 50 mL of LB media with appropriate antibiotics (streptomycin 100 μg mL^-1^ and gentamicin 40 μg mL^-1^) and grown until an OD_600_ of 1.0 was achieved. On the day of conjugation, the conjugation plates (20 mL, yeast synthetic complete medium lacking histidine 1.8% agar and 10% LBmc media) were made and the *S. meliloti* and *S*. *cerevisiae* cells were thawed on ice for approximately 20 minutes. Once thawed, 50 µL of *S. cerevisiae* was added to 100 µL of *S. meliloti* and mixed by gentle pipetting before being transferred to the plate and spread evenly. The plates were incubated at 30°C for 3 hours. Next, the plates were scraped with 2 mL of sddH_2_O and mixed thoroughly by vortexing for 5 seconds. For each conjugation, 3 biological replicates and 1 technical replicate were used and 100 μL of each dilution (10^0^ – 10^-1^) for each sample was plated on selection plates (25 mL, yeast synthetic complete medium lacking histidine, 2% agar, supplemented with ampicillin (100 µg mL^-1^)). The plates were incubated at 30°C, were scored after 4 days, and conjugation frequency was calculated.

**1.1.3. Conjugation from *E. coli* to *S. cerevisiae* – *Cis-* and *Trans*- Configuration**

To assess bacterial conjugation of pSC5 to *S. cerevisiae* in *cis*-orientation two donor strains of *E. coli* harboring either pSC5 or pTA-Mob 2.0 (gentamicin 40 µg mL^-1^) were prepared as in **Methods and Materials 2.5** , and in *trans-* orientation two donor strains of *E*. *coli* harboring either pSC5 and pAGE2.0.T or pTA-Mob 2.0 and pAGE2.0.T (gentamicin 40 µg mL^-1^ and chloramphenicol 15 µg mL^-1^) were prepared as in **Methods and Materials 2.5** and all stored in the -80°C freezer. On the day of conjugation, the conjugation plates (*cis* – 20 mL, yeast synthetic complete medium lacking histidine 1.8% agar and 10% LB media; *trans* – 20 mL, yeast synthetic complete medium lacking tryptophan 1.8% agar and 10% LB media) were prepared and the *E. coli* and *S*. *cerevisiae* cells were thawed on the ice for approximately 20 minutes. Once thawed, 50 μL of *S. cerevisiae* was added into the *E. coli* tube containing 100 µL of cells and mixed by pipetting before being transferred to the plate and spread evenly. The plates were incubated at 30°C for 3 hours. Next, the plates were scraped with 2 mL of sterile double distilled water and mixed thoroughly by vortexing for 5 seconds. A dilution series (10^-0^ – 10^-7^) was generated and two technical replicates of 100 μL for dilutions 10^0^ – 10^-4^ were plated on selection plates (*cis* – 25 mL, yeast synthetic complete medium lacking histidine 2% agar supplemented with ampicillin (100 µg mL^-1^); *trans* – 25 mL, yeast synthetic complete medium lacking tryptophan 2% agar supplemented with ampicillin (100 µg mL^-1^)); and two technical replicates of 100 µL for dilutions for each sample (10^-4^ – 10^-7^) were plated on non-selective plates (25 mL, 1 × YPDA supplemented with ampicillin (100 µg mL^-1^)). The plates were incubated at 30°C, were scored after 4 days, and conjugation frequency was calculated.

**1.1.4. Conjugation from *E. coli* to diverse yeast and transconjugant analysis**

Conjugation proceeded as described in **Methods and Materials 2.5**, except once dried, the conjugation plates were incubated at 30°C for 12 hours and selection plates were incubated at 30°C for 3 days before the number of colonies was counted. To test the recovery of pSC5 plasmid from diverse yeast transconjugants, the plasmid was isolated from selected diverse yeast transconjugants. The recovered plasmids were transformed into *E. coli* by electroporation and re-conjugated back from *E. coli* to diverse yeast species following the protocol in **Methods and Materials 2.5**, except on selective plates spot plating rather than full plates were used. After conjugation, cells were scraped with 2 mL of sterile double distilled water. These cells were serially diluted in 96-well plates and 5 μL of different dilutions (10^0^ – 10^-4^) were spot plated in 1 × YPDA media supplemented with nourseothricin (100 µg mL^-1^).

**1.1.5. Conjugation-based Kill Assay in *S. cerevisiae***

To assess yeast killing facilitated by bacterial conjugation, three donor *E. coli* strains harboring pAGE2.0.T and either pSC5-toxic1, pSC5-toxic2, or pSC5-toxic3 (gentamicin 40 µg mL^-1^ and chloramphenicol 15 µg mL^-1^) and the recipient *S. cerevisiae* were prepared as in **Methods and Materials 2.5** and stored in the -80°C freezer. On the day of conjugation, the conjugation plates (20 mL, yeast synthetic complete medium lacking histidine 1.8% agar and 10% LB media) were made and the *E. coli* and *S*. *cerevisiae* cells were thawed on ice for approximately 20 minutes. Once thawed, 10 μL of *S. cerevisiae* was added into the *E. coli* tube containing 100 µL of cells and mixed by pipetting before being transferred to the plate and spread evenly. The plates were incubated at 30°C for 3 hours. Next, the plates were scraped with 2 mL of sterile double distilled water and mixed thoroughly by vortexing for 5 seconds. For each conjugation, 4 biological replicates and 1 technical replicate were used and 100 μL of each dilution (10^0^ – 10^-1^) for each sample was plated on both selective plates (25 mL, yeast synthetic complete medium lacking histidine, and yeast synthetic complete medium lacking tryptophan, 2% agar, supplemented with ampicillin (100 µg mL^-1^). The plates were incubated at 30°C, colonies were scored after 4 days, and killing efficiency (CFU on yeast synthetic complete medium lacking histidine / CFU on yeast synthetic complete medium lacking tryptophan) was calculated.
